# Combinatorial Approaches for Cancer Treatment Using Oncolytic Viruses: Projecting the Perspectives through Clinical Trials Outcomes

**DOI:** 10.3390/v13071271

**Published:** 2021-06-29

**Authors:** Alexander Malogolovkin, Nizami Gasanov, Alexander Egorov, Marianna Weener, Roman Ivanov, Alexander Karabelsky

**Affiliations:** Gene Therapy Department, Sirius University of Science and Technology, Olympic Avenue, 1, 354340 Sochi, Russia; Gasanov.nb@talantiuspeh.ru (N.G.); egorov.ad@talantiuspeh.ru (A.E.); weener.me@talantiuspeh.ru (M.W.); ivanov.ra@talantiuspeh.ru (R.I.)

**Keywords:** oncolytic virus, virotherapy, immune checkpoint inhibitors, cancer immunotherapy, clinical trials, cancer gene therapy

## Abstract

Recent cancer immunotherapy breakthroughs have fundamentally changed oncology and revived the fading hope for a cancer cure. The immune checkpoint inhibitors (ICI) became an indispensable tool for the treatment of many malignant tumors. Alongside ICI, the application of oncolytic viruses in clinical trials is demonstrating encouraging outcomes. Dozens of combinations of oncolytic viruses with conventional radiotherapy and chemotherapy are widely used or studied, but it seems quite complicated to highlight the most effective combinations. Our review summarizes the results of clinical trials evaluating oncolytic viruses with or without genetic alterations in combination with immune checkpoint blockade, cytokines, antigens and other oncolytic viruses as well. This review is focused on the efficacy and safety of virotherapy and the most promising combinations based on the published clinical data, rather than presenting all oncolytic virus variations, which are discussed in comprehensive literature reviews. We briefly revise the research landscape of oncolytic viruses and discuss future perspectives in virus immunotherapy, in order to provide an insight for novel strategies of cancer treatment.

## 1. Introduction

Immunotherapy is arguably the most rapidly evolving field of cancer treatment. Despite the long history of cancer being treated mainly by the “cut, poison and burn” principle, immunotherapeutics allowed to outsmart the tumor using the intrinsic potential of the immune system. The immune checkpoint inhibitors (anti-CTLA-4 and anti-PD-1) have been the first kind of drugs that gave patients hope and revolutionized the treatment of cancers previously thought to be incurable [1]. Since the establishment of immunotherapy approaches, dozens of immune checkpoint inhibitors were studied for the treatment of different cancers with various degrees of success [2,3]. The breakthroughs of immunotherapy had an avalanche effect and stimulated the research of multiple combinatorial approaches for cancer treatment [4,5].

The modern arsenal of immunotherapeutics is impressively rich and includes diverse drugs and approaches such as pro-inflammatory cytokines [6,7], cancer vaccines [8,9], adoptive T-cell therapies [10], antibody-based immunotherapies (bispecific T cell engagers, checkpoint inhibitors, antibody-drug conjugates) [11] and oncolytic viruses (OV) [12,13,14]. The latest is of particular interest because of the recent encouraging clinical results and extreme flexibility of virus development platforms.

The oncolytic potential of several viruses has been known for quite a while, however, its clinical application for cancer therapy has been hampered by a lack of knowledge in virus molecular biology and immune pathways [15]. Most OV show selective replication in tumor cells (have enhanced tumor tropism) and affect several key steps in the cancer–immunity cycle [16]. Factors that impair viral proliferation and boost the rate of viral clearance in healthy tissue (IFN type I response genes, tumor suppressor proteins, etc.) are dysfunctional in tumor cells, enabling preferential replication of OVs [17,18,19]. Although some viruses exhibit inherent tropism for tumor cells, genetic engineering also helps to precisely tune up the virus life cycle to become the most potent cancer killer [20,21,22,23]. An arsenal of OVs is continually replenishing and nowadays predominantly represented by eight families: *Adenoviridae, Herpesviridae, Poxviridae, Picornaviridae, Paramyxoviridae, Rhabdoviridae, Parvoviridae* and *Reoviridae.*

The therapeutic effect of OVs in cancer therapy is mediated by several main mechanisms that eventually lead to immunogenic cell death (ICD), a form of regulated cell death (RCD) [24]. Firstly, OV infection of cancer cells initiates direct cell lysis [25,26,27,28]. OVs can induce anti-angiogenesis through reduction of VEGF concentration, resulting in a loss of tumor perfusion that leads to apoptotic and necrotic tumor cell death [26,29,30]. OVs prime the apoptosis in cancer cells through the induction of endoplasmic reticulum (ER) stress mechanisms [31]. Importantly, OVs induce innate as well as adaptive immune responses. In particular, the lysis of tumor cells caused by oncolytic viruses leads to the release of danger-associated molecular patterns (DAMPs) such as uric acid, secreted adenosine triphosphate (ATP), high mobility group protein B1 (HMGB1), surface-exposed calreticulin (ecto-CRT), heat shock proteins as well as pathogen-associated molecular patterns (PAMPs) including viral components (nucleic acids, proteins and capsid components). Innate immune cells (DC and NK) recognize DAMPs and PAMPs and evoke an adaptive antitumor immunity [14,32,33,34,35]. The release of tumor-associated antigens and neoantigens (TAAs and TANs), which can be captured by tumor-infiltrating antigen-presenting cells (APCs) leads to T-cell response. It was reported that treatment of patients with advanced solid tumors using T-VEC, coxsackievirus or poxvirus (Pexa-Vec) increases levels of TAA-specific CD8 + and CD4+ T cells [30,36,37,38]. Understanding the ICD marker kinetics may provide vital information in regards to the efficiency of cancer therapy [39].

By May 2021, more than 200 clinical trials of diverse combinations of OVs and immunotherapies were found and many others are coming out on the regular basis. Oncolytic virus application is discussed in several immaculate reviews highlighting the results of completed clinical trials [18,19]. In this review, we will discuss combinations of oncolytic viruses and other cancer therapies currently studied in clinical trials with specific emphasis on their efficacy. 

## 2. Oncolytic Viruses in Clinical Trials 

### 2.1. Adenoviruses

Adenoviruses are a family of icosahedral, non-enveloped viruses that contain a double-stranded DNA genome and include four structural proteins (hexon, penton, fiber, and pIX), each of them contributing to the interaction with the host cell surface. These viruses are medium in size (70–90 nm) [40]. There are 57 serotypes of adenoviruses are known so far. Adenoviruses possess some advantageous characteristics for cancer gene therapy, such as high efficiency of gene transfer in both dividing and non-dividing cells, a low risk of insertion mutagenesis, and replication in an exponential manner (having entered an infected cell, one virus can produce more than 10,000 progeny viruses). They have a wide range of tissue tropism and relatively large DNA loading capacity (up to 8.5 kb DNA in the case of adenovirus that contains a DNA genome of 36 kb) [41].

After more than 40 years of research, it is not surprising that adenoviruses have been thoroughly tested as vaccines, gene delivery vectors and oncolytics for many clinical applications. We found 30 registered clinical trials where oncolytic adenoviruses are used either alone (20 trials) and in multi-agent cancer therapy (10 trials). Recombinant unarmed adenoviruses demonstrate low toxicity (Grade 1–2) and impressive oncolytic potential which can be boosted by additional expression of immunostimulatory molecules (IFN-gamma, GM-CSF) or p53 (rAd-p53) [42,43,44]. More than 400 patients have been treated with rAd-p53, mainly in combination with chemotherapy [45], radiotherapy [46,47,48,49,50,51,52,53] and tumor resection [54] with various efficacy. Thus the overall response rate for combined treatment with cisplatin of malignant pleural effusion caused by lung cancer has reached an encouraging 82% [50].

Three adenovirus-based therapeutics have reached Phase III clinical trials (rAd-p53, rhAd5, Enadenotucirev En (rAd-HSV-1 TK)) [51,52,53,54,55]. Among them, human recombinant adenovirus carrying the p53 gene (rAd-p53) seems to show firm efficacy with mild adverse effects in combination with chemotherapy for advanced oral carcinoma (ChiCTR-TRC-09000392). Despite the fact that a large variation in the follow-up length from 3 to 86 months (36 months median) was observed among the advanced stages carcinoma patients, primary lesions in 58 out of 92 patients showed response to therapy [42]. The synergistic effect of rAd-p53 with chemotherapy (cisplatin) was also assessed for lung cancer treatment. The authors declare a higher efficacy rate of rAd-p53 therapy compared with cisplatin therapy (71.26 vs. 54.47%) [55]. The readers refer to the recently published systematic review highlighting the progress of the rAd-p53 therapy [56]. 

Recombinant Adenovirus-based (rhAd5) Oncorine (H101) was approved by Chinese authorities for nasopharyngeal carcinoma treatment in combination with chemotherapy in 2005 [57]. Oncorine has undergone several multi-center studies and has demonstrated prominent anti-cancer efficacy (mainly squamous cell carcinoma of the head and neck cancer (SCCHN) and nasopharyngeal cancer (NPC)) combined with chemotherapy. The manufacturer declared high response rate (over 78%) after 21 day cycle of combined treatment with rAd5 (5.0 × 10^11^–1.5 × 10^12^ per day), 5-FU (500 mg/m^2^) and cisplatin (20 mg/m^2^) (http://www.sunwaybio.com.cn/en/product.html, acessed on 10 May 2021). Oncorine’s historical overview with an update of clinical trials is summarized by Min Liang [57].

Oncorine brother-in-law ONYX-015 (CI-1042, d11520) is a chimeric human group C adenovirus (Ad2 and Ad5) with altered expression of the E1B gene. Injections of ONYX-015 were well tolerated and demonstrated improved outcomes in patients with accessible head and neck cancer. The results of the Phase II trial with intratumoral administrations of ONYX-015 showed 14% partial complete regression, disease stabilization in 41% and disease progression in 45% [58]. However, further clinical testing of ONYX-015 was interrupted. The exact mechanisms of selective anti-tumor activity of recombinant adenoviruses used in either Oncorine or ONYX-015 remain to be understood.

Enadenotucirev (ColoAd1/EnAd/chimeric Ad11/Ad3) was initially selected for its ability to mediate cancer cell death primarily in colorectal sites. Enadenotucirev also elicits high potency for the systemic treatment of metastatic or advanced epithelial tumors [59]. The results of Phase I/II trials (NCT02028442 (EVOLVE), NCT02028117 (OCTAVE)) have allowed determining the maximum tolerated dose, tolerability and safety profile of Enadenotucirev treatment [60]. Only one study (NCT02636036 (SPICE) is currently (Enadenotucirev (rAd-HSV-1 TK) assessing the safety and tolerability of Enadenotucirev in combination with nivolumab in 135 patients with metastatic or advanced epithelial tumors.

Adenovirus (Ad5) encoding a cytosine deaminase/HSV-1 thymidine kinase (Ad5-CD/TKrep) accompanied by prodrug (ganciclovir) administration render malignant cells sensitivity. Delivery of suicide genes into cancer cells sensitizes them to radiation. Patients with high-grade glioma recruited in Phase III trial (ASPECT) demonstrated a longer median time to death (308 days) than in the control group (268 days). Nevertheless, no difference in terms of overall survival was observed [47]. 

### 2.2. Herpesviruses

Herpesviruses constitute a large family of DNA viruses, which infect both animals and humans. HSV virions (150–240 nm) have a complex multilayered structure packaging massive DNA (~130–250 kb depending on the virus type) [61]. Currently, more than 130 herpesviruses are known. Human herpesviruses include herpes simplex viruses 1 and 2, Epstein–Barr virus, varicella-zoster virus, cytomegalovirus, Kaposi’s sarcoma-associated herpesvirus, human herpesviruses of type 6 and 7. Herpes simplex virus 1 modified by genetic engineering is of particular interest for oncolytic therapy [62,63,64,65].

Human herpes virus type 1 (HSV-1) is arguably one of the best commonly known viruses, affecting around 67% (3.7 billion) of the human population worldwide [66]. HSV-1 application for cancer therapy was meticulously tested in 39 clinical trials (see Supplementary Material). Many types of genetically engineered attenuated HSV-1 have been developed to utilize its intrinsic oncolytic properties (G207, 1716, OncoVEX, NV1020, HF10, G47) [67]. Talimogene laherparepvec (T-VEC, Imlygic, Oncovex-GM-CSF) holds the status of the one and only US FDA-approved oncolytic virus [19]). T-VEC was also approved by the Australian Therapeutics Goods Administration and European Commission for unresectable IIIB, IIIC and IVM1 stage melanoma. Genetically attenuated HSV-1 (JS1 strain) based T-VEC expresses human GM-CSF that is an essential mediator of dendritic cells recruitment, maturation and survival. Randomized Phase III clinical study (OPTiM) enrolled 436 patients with stage IIIB/C and IV melanoma and demonstrated an impressive durable response rate (DRR) (25.2% versus 1.2% in the group treated with GM-CSF only). Median overall survival (OS) was 41.1 months with T-VEC compared with 21.5 months with GM-CSF alone [19,68,69]. The most recent retrospective analysis of 88 patients with melanoma treated with T-VEC in Austria, Switzerland and Germany has demonstrated a high ORR (63.7%) and substantial complete remission (CR) rate (43.2%) [70].

Immune checkpoint inhibitors (ICI) as new anticancer medicines have shaped the trajectory of OVs clinical development. A combinatorial approach using oncolytic viruses and ICI may become a new paradigm for the treatment of tumors not responding to ICI. T-VEC was studied in combination with anti-PD-1 monoclonal antibody pembrolizumab (NCT02263508) and anti-CTLA4 ipilimumab (NCT01740297) in 713 and 217 patients, respectively. Durable response rate (13% versus 29.6%) was observed in an open-label Phase II study in patients with melanoma treated with ipilimumab (3 mg/kg every 3 weeks) or with a combination of T-VEC and ipilimumab (10⁶ PFU/mL every two weeks and 3 mg/kg of ipilimumab). Despite the fact that the objective response rate (ORR) with combination therapy was higher (38.8% versus 18%), progressive disease (PD) was observed in a similar number of patients (31% and 33% patients, respectively) [71].

Blocking the PD-1–PD-L1 interaction leads to the re-activation of T-lymphocytes and modulates their anticancer activity. For example, a combination of pembrolizumab with T-VEC in Phase III clinical trials demonstrates doubling of the response to therapy compared to the use of T-VEC alone [2].

One Phase I study of T-VEC combined with nivolumab was recently terminated due to slow accrual and withdrawal of funding (NCT03597009), but two studies are continuing. NCT02978625 is a Phase II trial of T-VEC combined with nivolumab in treating patients with refractory lymphomas or non-melanoma skin cancers, and the second study (NCT04185311) explores the safety and efficacy of T-VEC in combination with nivolumab and ipilimumab administered before surgery in breast cancer patients. 

The combination of OV with nivolumab seems to be quite a promising strategy. Thus preliminary results of herpesvirus-based OV armed with GM-CSF, pseudotyped by envelope fusogenic glycoprotein of gibbon ape leukemia virus (GALV-GP-R) [72] and combined with nivolumab showed that three of the four anti-PD1 refractory melanoma patients were responding to treatment, as were five of the six non-melanoma skin cancer patients (with three complete responses). Tumor biopsies showed immune activation, including recruitment of CD8+ T cells and increased PD-L1 expression. Moreover, the treatment was well tolerated as expected (Grade 1–2 adverse events) [73].

### 2.3. Poxviruses

*Poxviridae* family is a highly diverse and significant group of relatively large viruses able to attach and enter a wide variety of both vertebrate and invertebrate cells. About 22 genera of *Poxviridae* are known, among which four genera can infect humans: *orthopoxvirus, parapoxvirus, yatapoxvirus,* and *molluscipoxvirus*. Their virions are generally enveloped and have a shape of brick or oval form. Poxviruses feature a large size of about 200 nm in diameter and 300 nm in length. The genome contains a single, linear, double-stranded DNA. Vaccinia virus as one of the most commonly used poxviruses is suited for large transgene insertion due to the large size of its double-stranded DNA (more than 190 kb) [74]. Possessing a rapid replication and infection cycle vaccinia viruses cause cell lysis within 12–48 h [75]. These properties make vaccinia attractive for oncolytic virotherapy. The vaccinia variant Pexa-Vec is currently undergoing clinical trials in combination with ipilimumab, durvalumab or nivolumab.

Poxviruses are the most widely studied oncolytic viruses in relevant clinical applications. The history of experimental virology began from smallpox, variolation was the first documented therapeutic use of attenuated virus for disease prevention. Currently, non-human poxviruses are used to target tumor cells, following the footsteps of Edward Jenner, who was the first to introduce the cowpox virus as an experimental vaccine. By now there are six poxviruses from four different genera investigated as potential oncolytics: *Vaccinia virus* (VV), *Racoonpox* and Cowpox virus (*Orthopoxviruses*), Myxoma virus (*Leporipoxvirus*), Yaba monkey tumor virus (*Yatapoxvirus*), and Squirrelpox virus [76]. 

Historically, the vaccinia virus is the most utilized for therapeutic purposes—it was used for decades by the World Health Organization for the vaccination program that led to mass eradication of smallpox and created a well-established safety record. Initial clinical trials utilized wild-type vaccinia strains, specifically Dryvax that was first licensed by the FDA in 1931, and its derivative ACAM2000 that is currently licensed in the U.S. The study of the Dryvax strain of vaccinia included bladder cancer patients that received increasing doses of intravesical virus injections followed by cystectomy. Results of this study confirmed the safety of intratumoral vaccinia virus injection and its ability to induce local inflammatory response [77]. In the recent clinical study patients with solid cancers and acute myeloid leukemia received injections of autologous stromal vascular fraction cells infected by the ACAM2000 strain of vaccinia. Despite a trend towards longer survival demonstrated by few patients with evident oncolytic virus activity, the difference between survival curves was not statistically significant [78].

Additional attenuation of virus strains can be achieved by inactivation of viral growth factor and thymidine kinase genes (e.g., in vvDD recombinant vaccinia virus), which results in replication deficiency and inability to replicate in non-dividing cells. The vvDD (JX-929) was tested in Phase I clinical trial in 16 patients with advanced solid tumors. Intratumoral injections in patients with different cancer types (melanoma, colon, breast and pancreatic cancer) demonstrated no dose-limiting toxicities, thus a maximum feasible dose of 3 × 10^9^ PFU was defined. Remarkably virus replication occurred not only in classic vaccinia necrosum lesions, but also in non-injected tumors [79]. Quite possibly secondary infection of new tumors occurred was due to systemic vvDD spread. Safety of intravenous injections of vvDD was confirmed in Phase I clinical trial conducted in 11 patients with advanced solid tumors. However, median survival was just 4.8 months (range 2.6–23.9 months) and only one patient demonstrated mixed response: PET-CT imaging demonstrated complete resolution of newly formed hepatic metastases six weeks after treatment [80]. 

GL-ONC1 is yet another vaccinia-based OV drug developed by *Genelux company*. Significant changes, such as insertion of three expression cassettes encoding a fusion of Renilla luciferase and Aequorea GFP, beta-galactosidase (lacZ), and beta-glucuronidase replacing, respectively, genes of virulence factor F14.5L, thymidine kinase J2R and hemagglutinin A56R in the viral genome, affected its ability to replicate in non-dividing cells and made it possible to detect infection [81]. The GL-ONC1 was studied in seven completed Phase I (I/II) clinical trials with total of 168 cancer patients encompassing a wide range of pathological conditions [82]. Use of different administration methods, including systemic intravenous and local (intratumoral, intrapleural, intraperitoneal) GL-ONC1 administration, both as a monotherapy or in combination with conventional therapy, demonstrated the absence of significant adverse events.

Further studies using recombinant vaccinia virus armed with GM-CSF gene, the lacZ under the control of viral promoter p7.5. and viral thymidine kinase gene deletion (JX-594, pexastimogene devacirepvec, Pexa-Vec) has demonstrated limiting replication in cells with high intrinsic thymidine kinase activity characteristic for tumor. Pexa-Vec was assessed in clinical trials enrolling patients with multiple types of cancer. A dose-finding Phase II study was performed in advanced hepatocellular carcinoma (HCC) patients [83]. Infusion of the higher viral dosage resulted in a statistically significant improvement of overall survival compared to the group receiving the lower dose. The median overall survival was 14.1 months in the high-dose group and 6.7 months in the low-dose group. HCC patients treated with Pexa-Vec alone demonstrated a 15% response according to modified RECIST criteria. However, an open-label randomized Phase IIb TRAVERSE trial of Pexa-Vec in the second-line HCC patients after multikinase inhibitor sorafenib failure did not reach its primary survival endpoint (NCT01387555, [84]). Global double-blind Phase III PHOCUS trial evaluating Pexa-Vec followed by sorafenib treatment and versus sorafenib in a cohort of patients with advanced unresectable hepatocellular carcinoma initiated at the end of 2015 was terminated because it was unlikely to demonstrate an increase in overall survival (NCT02562755). 

Pexa-Vec Phase II trial in patients with metastatic refractory renal cell carcinoma who failed at least one prior VEGF/R-targeted therapy demonstrated a 76% RECIST disease control rate at Week 6 including one complete response [85].

It needs to be mentioned that Pexa-Vec is currently studied in several ongoing clinical trials: a Phase I/II trial assessing a combination of the oncolytic virus with nivolumab in first-line treatment of advanced HCC (NCT03071094), Phase Ib/IIa of Pexa-Vec in combination with cemiplimab for metastatic or unresectable renal cell cancer (NCT03294083), a Phase I trial of combination with ipilimumab in solid tumors (NCT02977156), a Phase I/II of combination with immune checkpoint inhibitors durvalumab and tremelimumab in refractory colorectal cancer (NCT03206073), and as the neoadjuvant therapy [86]. 

In addition to a combination of OVs with ICI as distinct agents, there is another combinatorial strategy tested in clinical studies—the insertion of genes encoding for immunomodulatory proteins into the virus genome. PROSTVAC (Rilimogene galvacirepvec/rilimogene glafolivec) is a poxvirus-based neoadjuvant vaccine developed for treatment of all prostate cancer types, specifically advanced cases of metastatic castration-resistant prostate cancer (mCRPC). It consists of two different poxvirus strains (vaccinia and fowlpox) expressing PSA (prostate-specific antigen) and TRICOM, a triad of costimulatory molecules (B7-1/ICAM-1/LFA-3) that enhance antigen presentation and activate cytotoxic T-cells to trigger the immune system in response to PROSTVAC injection. Although PROSTVAC was generally well-tolerated and demonstrated positive clinical results in the Phase II study, global double-blind Phase III (NCT01322490) PROSPECT study (1749 mCRPC patients) failed to validate the effect on overall survival or AWE (patients alive without events) endpoints [87]. Another clinical study of a similar therapeutic vaccine, carrying the TBXT gene coding brachyury protein instead of PSA, is ongoing. This product targets the epithelial–mesenchymal transition in order to prevent metastatic progression [88]. 

### 2.4. Enteroviruses (Picornaviridae)

Picornaviruses are a large family of small, positive-sense, single-stranded RNA viruses with a 30 nm icosahedral capsid. This family includes related non-enveloped RNA viruses, which infect vertebrates including mammals and birds. More than 300 types of enteroviruses have been discovered demonstrating incredible variability and heterogeneity [89]. Coxsackievirus [17,90], Enteric Cytopathogenic Human Orphan (ECHO) serotype 1, 7 and 12 [91,92] and chimeric poliovirus type 1 modified with human rhinovirus IRES region (PVS-RIPO) [93,94] are among the most well-studied in clinical trials. 

Coxsackieviruses are placed into two A and B, with serotype A21 being most commonly used because of its cytotoxicity against cancer cells [95]. Other serotypes, such as A13, A15, and A18, were also studied for oncolytic activity [96]. Coxsackievirus A21 (CVA21) exhibited potent oncolytic activity against myeloma xenografts. The Coxsackievirus variant CAVATAK in combination with ipilimumab and pembrolizumab is undergoing clinical trial [97].

Another representative of this virus family, poliovirus (*Enterovirus* genus), which is responsible for paralytic poliomyelitis, also can be applied in virotherapy. Poliovirus infection is rapid and releases about 10,000 mature virions per infected cell within 6 h after infection [98]. However, the use of wild-type poliovirus is associated with neurotoxicity. To minimize it, the neuroattenuated type of poliovirus, PVS-RIPO, was developed. It was studied in grade IV malignant glioma with no neurotoxicity reported [99].

Enteroviruses’ oncolytic potential was first shown in the 1950s [100]. One of these peculiar examples is the discovery of oncolytic enteroviruses made by Dr. Voroshilova at the Institute of Poliomyelitis and Viral Encephalitis in 1960–1970′s. Massive screening for children’s fecal samples during the polio eradication campaign in the late 1950s resulted in the discovery of hundreds of non-pathogenic enteroviruses [101,102,103]. Virologists have tested their ability to induce an immune response against pathogenic enteroviruses and revealed their remarkable efficiency [104]. Intriguingly, some enteroviruses have also demonstrated a peculiar feature in interfering with tumor progression [105,106]. Voroshilova’s successors have elaborated further on this finding and exploited Coxsackievirus, ECHO type 1 and ECHO type 2 viruses and type 1 poliovirus as oncolytic agents [107]. Coxsackievirus B6 (LEV-15L strain) has been patented as an efficient treatment against HPV-negative cervical cancer (RU 2496873). Coxsackievirus A7 and B5 have been successfully used to destroy glioblastoma stem cells and reduce tumor development [11,108]. In order to minimize immune response after oncolytic virus administration, a novel delivery system using human dendritic cells (US2020/0352993) and NK-92 cells has been developed [109].

Another example of oncolytic enterovirus with pronounced oncolytic properties is ECHO type 7 [110]. After additional directed evolution and selection oncolytic ECHO-7 virus was later registered in Latvia in 2004 as Rigvir [94]. Several case reports demonstrate good tolerability and moderate efficacy of Rigvir in combination with surgery for the treatment of advanced melanoma [111,112]. Ongoing studies may reveal the true mechanism of action of ECHO-7 and broaden its therapeutic application [113,114].

Among 19 up-to-date clinical trials, unmodified Coxsackievirus (also known as CAVATAK, CVA21, V937) was used in 12 studies and PVS-RIPO was used in seven trials. The efficacy of monotherapy using either CAVATAK or PVS-RIPO has been evaluated in six clinical trials (NCT03564782, NCT02986178, NCT03712358, NCT04577807, NCT03043391, NCT01491893). The main cancer types, which have been treated with enteroviruses are melanoma, malignant glioma and glioblastoma [94,115]. Remarkably, none of these clinically-tested enteroviruses carried any transgene. 

Despite the higher level of pre-existing immunity to Coxsackievirus A21 [116], it became the most intensively used oncolytic picornavirus because of its intrinsic affinity to various tumor cell types expressing intercellular adhesion molecule I (ICAM-1). CAVATAK-based oncolytic immunotherapy includes combinations with pembrolizumab (6 trials) and ipilimumab (2 trials) (NCT02307149, NCT02307149, NCT04303169). Intravenous administration of CAVATAK to patients with various solid cancers has demonstrated no Grade 3–4 product-related adverse effects. Additionally, CAVATAK combined with pembrolizumab in advanced NSCLC and bladder cancer was generally well-tolerated with no dose-limiting toxicities (NCT02043665).

Phase I clinical trial of PVS-RIPO in recurrent glioblastoma multiforme found survival rates to be significantly higher among treated patients. This stimulated further investigation and a Phase II trial of PVS-RIPO in combination with pembrolizumab was initiated recently (NCT04479241). 

Picornavirus from Seneca valley (Seneca valley virus, SVV-001, NTX-010) was first isolated as a cell culture contaminant [116]. Surprisingly, SVV-001 that does not infect normal human cells propagates in tumor cells of neuroendocrine origin. Phase I clinical trials using SVV-001 were conducted in a cohort of pediatric patients with neuroblastoma, rhabdomyosarcoma, or rare tumors with neuroendocrine features and demonstrated its moderate safety. However, SVV-001 was feasible and tolerable at the tested dose levels in the previous study, patients with extensive-stage small-cell lung cancer did not benefit from oncolytic virus treatment after chemotherapy with a platinum doublet. Quite the opposite, the presence of virus in the blood 1 or 2 weeks after treatment was associated with shorter progression-free survival [117].

### 2.5. Paramyxoviruses

Paramyxoviruses constitute large enveloped RNA viruses infecting mammals, birds, reptiles and fish. The family includes avian-specific Newcastle disease virus (NDV), epidemic parotitis virus (human), Sendai virus (human, mice), parainfluenza viruses 1–4, measles virus. Currently, 4 subfamilies, 17 genera and 78 species of paramyxoviruses are known. The genome contains a negative-sense, non-segmented RNA of 14.6–20.1 kb [118]. Viruses of this family replicate in the cytoplasm to form cytoplasmic or intranuclear inclusions in cells. Paramyxoviruses cause respiratory diseases, encephalitis, multiple hemorrhagic injuries of internal organs, conjunctivitis and general intoxication. Some paramyxoviruses, including attenuated strains of measles viruses, NDV, Sendai virus, possess oncolytic properties [27,119,120,121,122]. 

Newcastle disease virus (NDV), the avian oncolytic virus, has long been of interest to researchers as a potential anticancer agent, and clinical research on NDV has more than 50 years of history. Interest was sparked by the fact that attenuated NDV strains have been used for decades to prevent Newcastle disease in birds and that the virus is unable to cause serious illness in humans. As a result of the studies of different NDV strains for the presence of oncolytic activity, both non-lytic and oncolytic strains were described [123]. The first results of using NDV to treat a patient with acute leukemia were published in 1964 [123]. To date, we found 19 clinical trials where NDV is used as mono (6 trials) and multi-agent cancer therapy (13 trials). Interestingly, most of the clinical trials with NDV were completed in the late 1990s with only two registered (see Supplementary Material). 

The following approaches were used to apply NDV for cancer treatment:

1. NDV oncolysate vaccines [124,125,126];
2. autologous tumor cell vaccine (ATV-NDV) [127,128,129];
3. oncolytic NDV alone and in combination with durvalumab (NCT03889275, NCT04613492) [130,131].

Oncolytic strain 73 T was first reported in 1965 and has been used in several clinical trials for the treatment of cancer patients by NDV oncolysate vaccines [132]. In two Phase II clinical trials involving 83 patients with Stage II metastatic melanoma, NDV oncolysate was used as an additional immunotherapeutic agent. A 10-year follow-up of these 83 patients showed that 60% were alive and free of recurrent disease [124]. At 15-years of follow-up, 55% were alive [124]. A Phase II study of NDV 73 T autologous oncolysates has been conducted involving 208 patients with locally advanced renal cell carcinoma [124]. In this study, interleukin-2 and interferon-alpha were added to oncolysate vaccines. The results showed an improvement in disease-free survival (DFS) compared to published survival data for similar patients who received surgical treatment only. 

In addition to the use of NDV in oncolysate vaccines, native unarmed strains as PV701, MTH-68/H, and HUJ were administered for the treatment of various types of cancers [132,133,134,135]. 

Phase I trial of PV701 in 79 patients with advanced solid cancers was designed to define the maximum-tolerated dose (MTD) and safety of PV701 as a single agent by single and multiple intravenous doses administration. The researchers suggested that PV701 warrants further study as a novel therapeutic agent for cancer patients [133]. 

Another NDV strain (HUJ) was evaluated in the Phase I/II trial in 14 patients with recurrent glioblastoma multiforme (GBM). The virus was administered intravenously for 15 min and was well tolerated. The toxicity was minimal, five patients had Grade 1–2 fever. The maximum tolerated dose was not achieved. One patient achieved a complete response, while others developed progressive disease. The researchers concluded that good tolerability and encouraging responses warrant further assessment of NDV (HUJ strain) in GBM, as well as in other types of cancer [132]. 

In an open Phase II/B, placebo-controlled clinical trial (26 patients), another attenuated NDV strain (MTH-68/H) was administered by inhalation for the treatment of 33 patients with lung metastases. Patients receiving viral therapy had high survival rates after 2 years. Only 7 out of 33 patients survived, while none survived in the control group [134]. Encouraging results were also obtained in another clinical trial with the administration of MTH-68/H for the treatment of patients with advanced high-grade glioma. Four patients were treated with MTH-68/H after traditional anticancer therapies proved ineffective. This treatment resulted in a life span of 5–9 years and improved quality of life for each patient. Notably, this disease has a poor prognosis with a life expectancy of six months to a year [135]. 

In 1979, Kobayashi (Chiba, Japan) described that a live cell vaccine is more immunogenic than a lysate and developed the term “tumor xenogenization”, which led to the concept of using a live cell vaccine rather than lysate [136]. Later, several in vivo studies have shown the efficacy of NDV-modified autologous tumor cells for anticancer therapy [137,138] and also that NDV-modified autologous tumor cells augment the tumor-specific T cell response [139,140]. Based on this concept, an autologous tumor cell vaccine modified by infection with NDV (ATV-NDV) was developed [130]. We found eight clinical trials where ATV-NDV is used as cancer therapy (see Supplementary Material). ATV-NDV promotes immunogenic cell death and systemic anticancer immunity. Clinical experience with ATV-NDV demonstrates a good safety profile [129,141,142,143,144]. In addition, ATV-NDV clinical trials showed promising results for the treatment of early breast cancer, metastatic breast cancer, metastatic ovarian cancer [127], head and neck squamous cell carcinoma (HNSCC) [142], resected colorectal carcinoma [128], advanced renal-cell carcinoma [143], GBM [145], metastatic colon cancer [146]. There is a significant potential for using NDV in combination with other therapies, such as immune checkpoint inhibitors (ICI). Two clinical trials using the combination of attenuated NDV carrying GM-CSF gene (MEDI5395) and IL-12 gene (MEDI9253) with durvalumab (anti-PD-L1) are ongoing (NCT03889275; NCT04613492).

Sendai virus strain Moscow (SeVM) has been of particular interest for tumor treatment since the 1960s when chicken-embryo adapted strain was used as an alternative therapy for progressive tumor cases. Much later, in 1995, several small-scale clinical studies with patients with advanced tumors (stage II and IV) were conducted using SeVM. In some cases, SeVM-based therapy slowed down tumor progression with long-term remission [147,148]. Oncolytic SeVM strain was later sequenced and deposited in Genbank (KP71417) [149]. The method and composition for cancer immunotherapy based on the oncolytic non-pathogenic SeVM strain have been patented. The recent application of the SeVM strain in primary prostatic adenocarcinoma cell lines has also demonstrated promising results and broadened the oncolytic potential of this virus [150].

Attenuated measles virus (MV), which belongs to *Morbillivirus* genus, is another representative of oncolytic paramyxoviruses. The oncolytic properties of MV have been studied for more than a decade [151,152]. So far, we found 10 clinical trials where MV is administered for mono (seven trials) and multi-agent cancer therapy (three trials) (see Supplementary Material). The first clinical testing of an unmodified live MV (Edmonston–Zagreb vaccine strain) as an oncolytic agent was a dose-escalation Phase I trial performed by Heinzerling et al. [146]. A total of 16 injections of MV were administered intratumorally to five measles-immune patients with cutaneous T-cell lymphoma (CTCL), which were pretreated with interferon-alpha to prevent uncontrolled virus spread. The study showed that the well-tolerated treatment with MV induced the characteristic cytopathogenic effect of viruses on tumor cells and resulted in clinical responses despite the presence of preexisting neutralizing antibodies (NAbs). Tumor regressions were observed in three out of five patients. Interestingly, improvement also occurred in distant non-injected lesions in two patients. 

To monitor the viral replication MV was genetically modified to express human sodium iodide symporter (hNIS) and human carcinoembryonic antigen (CEA). The Mayo Clinic (USA) has initiated numerous Phase I/II clinical trials to investigate the clinical safety and utility of MV-CEA and MV-NIS (NCT00390299, NCT02364713, NCT02068794, NCT02700230, NCT01503177, NCT01846091). 

The first clinical trial of intraperitoneal administration of MV-CEA was conducted in 21 measles immune patients with platinum- and paclitaxel-refractory ovarian cancer [153]. The dose-dependent CEA elevation in peritoneal fluid and serum was detected, while dose-limiting toxicities were not observed at any dosage levels. All observed adverse effects were Grade 1 and 2, the most common of which were fever, fatigue, and abdominal pain. The median overall survival (OS) was 12.15 months, while the expected median OS in this patient population is 6 months [153].

Another Phase I trial of intraperitoneal MV-NIS administration in patients with paclitaxel and platinum-resistant ovarian cancer evaluated the safety of MV-hNIS and the utility of NIS-based imaging for monitoring of viral gene expression in malignant cells [154]. Similar to the previous MV-CEA trial, in this study, the absence of dose-limiting toxicity and mild Grade 1 and 2 adverse effects were observed. Median OS was 26.6 months, which compares favorably with OS (6–12 months) observed in other trials targeting the same platinum-resistant patient population [154]. 

The overall clinical experience with both an unmodified MV and recombinant MV in patients suffering from various types of cancer is highly encouraging. Expression of soluble marker CEA by recombinant MV provides an opportunity to assess viral replication. The expression of the hNIS gene can promote the accumulation of radioactive iodine isotopes in cancer cells. This in turn allows to image viral gene expression in malignant cells, as well as provides an opportunity for radiotherapy [154,155]. 

### 2.6. Rhabdoviruses

*Rhabdoviridae* family currently includes 30 genera [92]. The virions of rhabdovirus consist of single-stranded RNA and have a width of about 75 nm and a length of about 180 nm [156]. VSV genome contains five genes: (1) N—nucleocapsid protein; (2) P—phosphoprotein; (3) M-matrix protein; (4) G—surface glycoprotein; (5) L—RNA-dependent RNA polymerase [157,158,159,160,161].

Rhabdoviruses cause infections in plants, invertebrates and vertebrates including humans. Several rhabdoviruses have been studied for oncolytic properties, among which vesicular stomatitis virus (VSV) and Maraba virus.

Vesicular stomatitis virus (VSV) seems quite promising as a safe and effective oncolytic virus, especially in combination with different immunotherapeutic approaches.

Low pathogenicity (the virus infects livestock animals, human infection usually goes asymptomatic), cytoplasmic replication (without integration into the genome), production of VSV in commonly used mammalian cell cultures (BHK, HEK) high viral titer, easy to modify genome, lack of pre-existing human immunity made VSV-based therapeutics an ideal platform for vaccines and oncolytic virus development [162,163,164].

Over the past decade, VSV has been successfully used to develop highly effective vaccines against infections, many of which are highlighted by WHO as a top priority [165] (“Blueprint list of priority diseases”)—Ebola, Marburg, Lassa, Nipah, Zika, CCHF, MERS, SARS, HIV [166]. In 2019 following Priority Review and Breakthrough Therapy designations the FDA approved pseudotyped VSV-ZEBOV vaccine against Zaire Ebola virus (Ervebo, Merck and Co., Inc.), based on the results of several clinical studies conducted in 2014–2016 (NCT02269423, NCT02280408, NCT02374385, NCT02314923, NCT02287480, NCT02283099, NCT02296983, NCT02344407, NCT02378753, NCT02503202), which confirmed high safety profile (based on monitoring of over 18,000 patients) and 100% prophylactic efficacy with only three cases of severe adverse events.

Compared to the vast amount of data on the VSV vaccine efficacy and safety, the number of clinical studies aimed at exploring the prospects for using VSV as an oncolytic virus is more than modest. However, at the same time, numerous preclinical studies are being conducted, demonstrating the high therapeutic potential of VSV-based oncolytics for the treatment of a wide range of tumors. Many of them are aimed at improving the oncoselectivity and mitigating oncotoxicity of VSV by replacing the G-protein (pseudotyping), genes shuffling, the addition of different payloads (tumor-suppressor genes, tumor-associated antigens, immunomodulators, reporter genes, miRNAs), by directed evolution of viral proteins, as well as by combining VSV with radiotherapy, chemotherapy, anti-angiogenic factors, immune checkpoint inhibitors, etc [167,168].

Currently, eight clinical trials are assessing VSV-based therapy for different types of cancer and four clinical trials evaluating Maraba virus-based therapy.

The only completed study of VSV to date (NCT03456908) evaluated the use of the sodium iodide symporter gene (hNIS) in the VSV or measles virus (MV) genome, respectively, and the F-18 tetrafluoroborate (BF4) as a PET (positron emission tomography) imaging biomarker for evaluating VSV and MV replication and virotherapy efficacy in two patients. Despite the lack of published data on the results of this study, there are seven ongoing additional clinical trials ((NCT02923466, NCT01628640, NCT03647163, NCT03865212, NCT03120624, NCT03017820, NCT04291105), in six of which hNIS is used as one of the payloads for VSVs.

Notably, in all eight clinical trials, hIFN-beta are used as one of the payloads to activate immune cells and increase tumor specificity by triggering the mechanism of IFN-dependent inhibition of viral replication in healthy cells [169,170].

Seven studies (NCT02923466, NCT01628640, NCT03647163, NCT03865212, NCT03120624, NCT03017820, NCT04291105) are in active phase, and preliminary results were found only for three of them. In particular, NCT02923466 is evaluating the safety and maximum tolerated dose (MTD) of VSV-IFNB-NIS in monotherapy for pheochromocytoma and neuroendocrine tumors and in combination with avelumab (anti-PD-L1 antibody) for colorectal cancer. According to the preliminary results from 18 patients in 3 groups after infusion of 1.7 × 10^10^ TCID50 VSV-IFNB-hNIS for 30/60/90 min in monotherapy (without avelumab), did not have serious adverse events (no DLTs, deaths or G3–4 related IRR AEs) or viral shedding. At the same time, PR/SD was observed in nine patients (50%), with a better response to therapy in case of 30-min infusion (five PR/SD out of seven patients). Upcoming is the efficacy study of VSV-IFNB-hNIS in combination with avelumab in patients with colorectal cancer [171]. The goal of the NCT03120624 dose-escalation study is the evaluation of the safety of VSV-IFNB-hNIS after IV infusion in combination with ruxolitinib (Jak inhibitor) for stage IV or recurrent endometrial cancer. An initial dose of 1.7 × 10^10^ TCID50 VSV-IFNB-hNIS was safe and well-tolerated (nine patients). Immuno-phenotyping of blood cells on days 3, 8, 15 and 29 after infusion showed that CD8 + T cells are activated (PD1 expression level was increased). Analysis of biopsies on days 29 and 3 months after infusion showed an increase in the number of TILs [172]. As for the Phase I clinical trial (NCT03017820) of intravenous administration of VSV-IFNβ-NIS for patients with relapsed refractory multiple myeloma, acute myeloid leukemia and T-cell lymphoma the published preliminary report showed that VSV-IFNβ-NIS is well tolerated at dose level 1.7 × 10^11^ TCID50. Although there was a response in T-cell lymphoma patients (one PR and one CR of four patients with TCL), there were no confirmed responses in patients with multiple myeloma. However, as the authors conclude, the observed reduction in serum level of involved free light chain (LC) following the infusion suggests that combinatorial approaches may be more effective in multiple myeloma patients [173].

Resistance to standard anticancer therapy, as well as resistance to ICI therapy, is the inclusion criterion in most clinical studies of VSV-IFNB-hNIS, which is typical for most oncolytics. An interesting exception is NCT03865212, which evaluates the safety and efficacy of VSV-IFNB-TYRP1 in the treatment of stage III-IV melanoma. Due to the lack of treatment standards for a rather rare uveal melanoma (ocular melanoma), such patients receive first-line virotherapy. TYRP1 (tyrosinase-related protein 1) is involved in melanin biosynthesis and is actively expressed in melanocytes. Despite the fact that functions and regulation of the protein are still not fully understood, TYRP1 is a known tumor-associated antigen and can be used to stimulate T-cell response [174,175,176].

The expected publication of results of several clinical trials in the next few years should reveal the prospects of using VSV not only in monotherapy, but also in combination with chemotherapeutic agents and checkpoint inhibitors. In particular, the Phase II study (NCT04291105) assesses the safety and efficacy of VSV-IFNB-hNIS in combination with cemiplimab (an anti-PD1 antibody that has been shown to be effective in the treatment of cutaneous squamous cell carcinoma) [177], with IV vs. IT administration for melanoma treatment, and with IV infusion for hepatocellular carcinoma, non-small cell lung cancer (NSCLC) and endometrial cancer treatment. The results have not been published yet. In a Phase I/II trial NCT03647163 the goal is to evaluate the safety and efficacy of VSV-IFNB-hNIS at a low dose (5 × 10^10^ TCID50) in patients with solid tumors resistant to standard therapy and at a higher dose (1.7 × 10^11^ TCID50) in combination with pembrolizumab in patients with head and neck squamous cell carcinoma (HNSCC) and NSCLC resistant to standard therapy and immunotherapy with anti-PD1/PDL1 antibodies. Phase I study NCT01628640 evaluates the safety and MTD of VSV-IFNB-hNIS in the treatment of hepatocellular cancer and tumors with metastatic lesions in the liver with two administration formats (injection into one or more lesions, respectively); and in Phase I study, NCT03017820 VSV-IFNB-hNIS is combined with ruxolitinib (JAK inhibitor) for the treatment of a wide range of refractory hematologic cancers.

As for the Maraba virus, out of four initiated clinical trials one was stopped (NCT03773744) due to insufficient drug supply. Two Phase I studies were focused on the safety and maximum feasible dose of Maraba Virus (MG1) expressing a conserved MAGE-A3 tumor antigen [178], in combination with the AdMA3 adenoviral tumor vaccine for the treatment of MAGE-A3 positive tumors (NCT02285816), and in combination with AdMA3 and pembrolizumab for the treatment of NSCLC (NCT02879760). In the 3rd study (NCT03618953) in patients with HPV-associated cancers, the safety and MTD of MG1 carrying two human papillomavirus (HPV) oncogenes E6 and E7 [179] is evaluated in combination with the Ad-E6E7 adenoviral vaccine and atezolizumab (anti-PD-L1). Unfortunately, the study results also have not been published yet.

### 2.7. Parvoviruses

Parvoviruses are a family of human and animal viruses with linear, single-stranded DNA genomes. Compared to many viruses, the parvovirus virion is small with a diameter of 23–28 nm. Its genome is enclosed in an icosahedral capsid that has a rugged surface. It is known that parvoviruses can replicate in tumor cells to cause their lysis [180,181,182].

The rat H-1 parvovirus (H-1PV strain) was shown to exhibit oncolytic activity against preclinical glioma models, through both direct oncolysis and stimulation of anticancer immune responses. Thus, a wild-type rat oncolytic parvovirus was chosen by *Oryx company* for further investigation. Two Phase I/IIa clinical trials of ParvOryx in recurrent glioblastoma (rGBM) (NCT01301430) and metastatic, inoperable pancreatic cancer were successfully completed (NCT02653313). The Phase I/IIa clinical trial (called ParvOryx01) in rGBM patients confirmed its safety and tolerability. The data provide a lack of evidence of ectotoxicity. Moreover, it was demonstrated that oncolytic parvovirus could pass the blood–brain barrier in both directions in a dose-dependent manner, supporting the possibility of systemic administration instead of intratumoral injections. Favorable progression-free survival was shown compared with historical controls—during the regular trial follow-up PFS at 6 months it was 27%, and median PFS was 111 days, whereas OS was approximately 72% and median OS was 464 days [183,184].

### 2.8. Reoviruses

*Reoviridae* family comprises 15 genera with 97 species. They may cause infections in plants, fish, birds, animals and humans. Some reoviruses such as rotaviruses and orthoreoviruses in particular, cause intestinal and respiratory infections in humans. Reoviruses have segmented double-stranded RNA genome (16–27 kbp in length) packaged in 55–85 nm non-enveloped virion [185,186]. The oncolytic potential of reoviruses was discovered more than 30 years ago. Like other oncolytics, reoviruses exploit altered signaling pathways (including Ras) in cancer cells that allow them to preferentially replicate at tumor sites [187,188,189,190,191].

Unmodified human Reovirus type 3 (Dearing strain) is one of the well-documented and tested oncolytic reovirus. We found 39 clinical trials of reovirus (also known as pelareorep, Reolysin) for the treatment of solid tumors and hematological malignancies. Pelareorep was used predominantly (31 out of 39 clinical trials) in combination with chemotherapy and radiotherapy. More than 1100 patients have been treated with pelareorep in combination with chemotherapy [192,193,194,195,196,197], radiotherapy [198] and polytherapy [199] with various effectiveness. Monotherapy with Reolysin as a single agent was found in eight studies.

It is worth noting that the most recent clinical trials on Phase I and II with Reolysin aiming at the evaluation of its anti-cancer efficacy in combination with ICI (8 studies, See Supplementary Material) are ongoing. The results of pelareorep combination with FOLFOX and bevacizumab (anti-VEGF antibody) show moderate tolerability with an increased ORR. However, no statistical support was observed in PFS and OS between the groups [200].

Pelareorep and anti-PD-1 therapy (pembolizumab) trial demonstrated encouraging efficacy in Phase I clinical trial with 11 patients enrolled. No severe toxicity was notified and disease control was achieved in three of the 10 efficacy-evaluable patients. Partial response was recorded for one patient for 17.4 months. Disease stabilization was observed in two patients, lasting 9 and 4 months, respectively [201].

Reovirus-based therapy was applied for metastatic colorectal, melanoma, sarcoma, adenocarcinoma, head and neck, pancreatic, prostate, glioma, NSCLC. Reoviruses demonstrated Grade 1–2 toxicity (flu-like symptoms) with sporadic 3–4 grade toxicity (neutropenia, respiratory failure). Pooled data analysis of the safety and tolerability of pelareorep from eight clinical trials are summarized by Gutierrez et al. [202]. Despite the long period of reovirus research, only one clinical trial has advanced to Phase III clinical trial testing efficacy of pelareorep in combination with chemotherapy in platinum-refractory head and neck cancers (NCT01166542). However, the results of this study are somehow very scarce. Evaluation of 105 patients with metastatic tumors, has resulted in tumor stabilization in 86% (n = 50), compared with 67% of patients (n = 55) in the control group [203].

## 3. Combination Therapies and Future Perspectives

**Figure 1:**
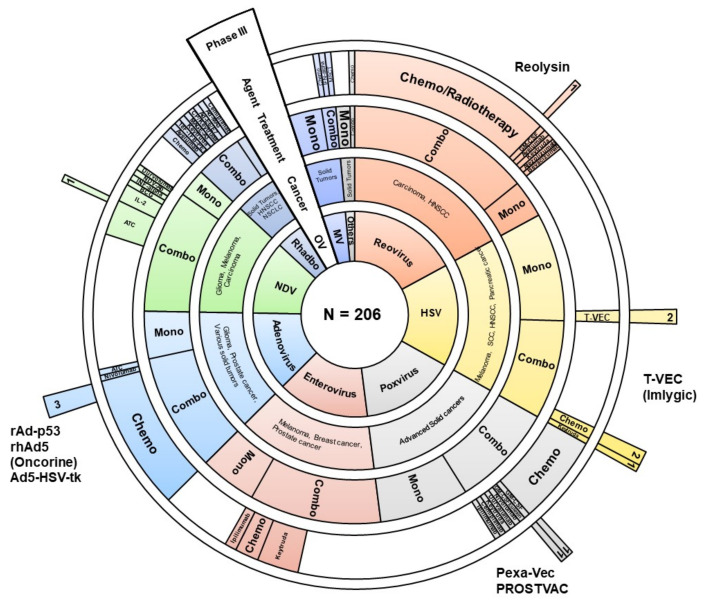
Graphical representation of 206 clinical trials using oncolytic viruses as an immunotherapy. Sunburst diagram depicts virus genus (inner circle), type of cancer, variants of therapy (single agent (mono) or combinatorial approach (combo), combinatorial agent (if applicable). Outer sector illustrates the drugs that have reached Phase III of clinical trials or have been registered. Chemo—chemotherapy, ATC—autologous tumor cells, HNSCC—head and neck squamous cell carcinoma, NSCLC—non-small-cell lung carcinoma, SCC—squamous cell carcinoma, BCG—*Bacillus Calmette–Guérin*, MSCT—mesenchymal stem cell therapy, GM-CSF—granulocyte-macrophage colony-stimulating factor, IL-2—interleukine-2 OV—oncolytic virus, HSV—herpes simplex virus, MV—measles virus, Rhabdo—rhabdoviruses (VSV and Maraba), NDV—newcastle disease virus, others—Parvovirus and Seneca valley virus.

In the past few years, we have seen an unprecedented increase in clinical trials using oncolytic viruses. Here, we reviewed 206 clinical trials found in open sources (databases and publications). For 109 studies, we managed to find relevant publications describing preliminary or final results (hereinafter, see Supplementary Material). Most of them are early-stage trial studies, with only 12 Phase III studies (Figure 1). Results of these clinical trials point to the limited efficacy of oncolytics despite all the advantages of subverted antiviral immune response in cancer cells.

Since oncolytic viruses can activate innate and adaptive immune mechanisms that are capable of targeting both cancer cells and viruses, it is very important to keep a balance between viral immunogenicity and anticancer immunity. One of the major causes of the limited efficacy of oncolytics observed in human trials is a pre-existing immunity against a virus. Antibodies to AAV [204], adenovirus [205], or enterovirus (ECHO, Coxsackievirus) are frequently found among cancer patients. According to literature among 391 healthy adults from 21 provinces and cities of China tested for the presence of antibodies 85.7%, 58.8% and 74.2% were found to be seropositive against enterovirus 71, coxsackievirus 16, and adenovirus human serotype 5, respectively [206]. It might be also the factor that hampers the efficacy of oncolytic HSV, since more than 67% of the population is latently infected by HSV-1 [66].

Intriguingly, pre-existing viral immunity is not always a hurdle for immunotherapy. Several promising preclinical models using oncolytic NDV and Adenovirus have successfully demonstrated that antibodies to the virus may potentiate anti-tumor immune response by retargeting antiviral antibodies and activating tumor-directed CD8+ T-cells [207,208]. In addition, anti-viral immunity may be exploited further to increase the efficiency of virus cancer therapy by rationale design of recombinant bifunctional adapter carrying the tumor-specific ligand and adenovirus immunogenic domain (hexon DE1) that activates tumor microenvironment, prolongs survival of mice and, of particular importance, can be possibly used to manage metastatic cancers [209]. Therefore, we do suggest that the pre-existing viral immunity is an important variable factor that may significantly influence the results of cancer virotherapy and should be critically accessed for a particular oncolytic virus.

**Figure 2:**
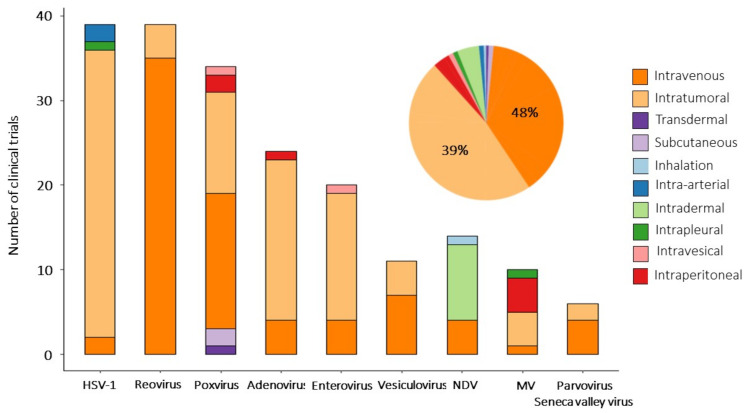
Routes of administration of oncolytic viruses. Bar plot shows delivery routes for every oncolytic virus used in reviewed clinical studies. Pie chart diagram summarizes the overall distribution of delivery strategies among clinical trials analyzed in this review.

To partly overcome the issue of preexisting immunity and to improve the efficacy of virotherapy use of plasmophoresis, immunosuppressants, non-human viruses and optimal delivery routes have been extensively studied [210]. OVs can be delivered systemically (intravenous injection) or directly (by intratumoral injection). Both delivery approaches have been applied in clinical studies, but intratumoral delivery was used predominantly. Clinical trials with H101 and talimogene laherparepvec have shown that intratumoral injections are the most effective and safe way to administer OV [211,212,213]. The intratumoral injection is a route of administration in 94 clinical trials (48%) and in 77 (37%) trials OVs are delivered systemically (Figure 2), wherein 10 studies assess the efficacy of IT vs. IV infusions (3 CTs—For VSV, 3—For poxviruses). The remaining 29 studies are exploring alternative routes of administration (intraperitoneal, intradermal, intrapleural, etc.) (see Supplementary Material). The abscopal effect, or systemic antitumor effect, has been registered after local administration of oncolytic viruses [214,215,216]. Virally induced oncolysis can promote the release of danger-associated molecular patterns that induce immune system awaking and systemic antineoplastic response. The abscopal effect can be boosted by the complementary use of oncolytics with active immunotherapeutic. For example, equivalent response rates were recorded in injected and distant tumors in patients with hepatocellular carcinoma treated with Pexa-Vec and GM-CSF [83]. Since not all tumor lesions are accessible for direct injection, especially in the case of metastatic disease, the systemic approach seems to be the preferable option. However, the rapid clearance of viruses from the bloodstream before they reach their targets reduces the efficacy of systemic administration [217,218,219]. Additional OV modifications (genetic engineering of capsids or chemical conjugation) may significantly expand the lifetime of OV circulation after systemic administration [213,220]. There is a need to confirm these findings in clinical trials.

The safety of OV therapy is an imperative question that should be addressed in any clinical trial. Encouragingly, most of the studies we reviewed report the safety of virotherapy (Grade 1–2) with only a few cases of severe adverse effects (Grade 3–4). Oncolytics are well-tolerated in various settings and cancer types [202]. An additional concern is the fact that viral infections may aggravate autoimmune conditions. It has been previously shown that B19 parvovirus may cause rheumatoid arthritis [221], coxsackievirus—type 1 diabetes [222]. Benefits of virotherapy may overcome potential risk for these patients; nonetheless requires careful investigation.

An additional boost is needed to counteract so-called “cold tumors” that do not respond well to immunotherapy. In this connection, the use of viruses in combination with ICI, immune-stimulating agents and chemokines (as separate agents or cloned into the OV genome) seems to be the most promising approach.

Combinatorial approaches become predominant in the treatment of cancers that are resistant to standard therapy or recurrent (117 clinical trials out of 206, 57%, Table S1 in Supplementary Material). Among the ICI the leading position is occupied by antibodies blocking the PD-1/PD-L1 axis, which bind either to PD-1 (pembrolizumab, nivolumab, avelumab) or to its ligand PD-L1 (durvalumab, cemiplimab, atezolizumab, HX008) (35 clinical trials out of 44, 80%), and pembrolizumab is used in most studies (14 studies).

The second most popular agent to combine with oncolytic viruses (vaccinia virus, HSV-1 and Coxsackievirus 21) is the CTLA-4 inhibitor (ipilimumab, tremelimumab) (6 studies out of 44, 13%). CTLA-4 inhibitors block the negative regulation of T-lymphocytes (B7-CTLA4 interaction), contributing to their long-term activation. Reassuring results indicate that the combination of talimogene laherparepvec with ipilimumab has greater efficacy than either therapy alone, without additional safety concerns above those expected for both medicines in monotherapy. Thus, 38 patients with melanoma (39%) in the combination arm and 18 patients (18%) in the ipilimumab arm had an objective response, and importantly, responses were not limited to injected lesions: responses in the visceral lesion were observed in 52% of patients in the combination arm and 23% of patients in the ipilimumab arm [3].

As for immunomodulatory agents, GM-CSF, IFN-alpha and IL-2 are most often used in combination with oncolytics (in nine clinical trials). Additional combinations of oncolytics are shown in Table 1.

Designing novel ICI is an extremely competitive area of research. The efforts are mainly focused on overcoming de novo or acquired resistance to certain ICI. Among the most promising candidates, several new molecules are currently undergoing preclinical/clinical trials (lymphocyte activation gene-3 (LAG-3), T cell immunoglobulin and mucin-domain containing-3 (TIM-3), T cell immunoglobulin and ITIM domain (TIGIT), V-domain Ig suppressor of T cell activation (VISTA), immune costimulatory molecule (ICOS) and its ligand (ICOS L) [223,224,225]. In particular, the multifaceted role of ICOS/ICOS L in T-cell activation is crucial for autoimmune diseases and cancer. We foresee that cancer virotherapy in combination with novel ICI might be a new powerful tool in the next-generation cancer immunotherapy.

The most commonly used payloads in oncolytic viruses are summarized in Table 2. The GM-CSF occupies a leading position among genes additionally integrated into the genome of oncolytic viruses (49 clinical trials, see Supplementary Material). The main role of GM-CSF is to stimulate the proliferation, differentiation and migration of macrophages and dendritic cells. The high pro-inflammatory and regulatory potential of GM-CSF promoted its use in the treatment of a wide range of oncological diseases [226,227,228,229].

The thymidine kinase (TK) gene of the herpes simplex virus HSV-1 is often cloned into the genome of oncolytic viruses (24 studies). Such modification allows mediating the death of TK-expressing cells, ultimately increasing the therapeutic potential of OVs mediated by antiviral drugs (ganciclovir) [47,64,229]. The gene encoding sodium iodide symporter (NIS) was used as a payload in the genome of VSV and measles virus (in 14 clinical studies) to visualize viral biodistribution and replication using CT and combination with radiotherapy. Other payloads include interferon-beta (INF-b) to improve oncoselectivity and stimulate the antitumor response, p53 to induce apoptosis, and MAGE-3 as one of the well-studied conservative tumor antigens—they were used in eight, six and three studies, respectively [42,171,230]. However, it should be noted that in 109 out of 206 clinical trials (53%), oncolytics viruses without any transgenes were used.

In this review, we discussed eight oncolytic virus families. However, this list is far from being complete. Mostly, we focused on the promising combinations based on the published clinical data, rather than presenting all oncolytic virus variations. Nevertheless, many preclinical and clinical studies using vaccines (e.g., yellow fever 17D strain [231]), attenuated (Zika virus [232]) or cancer cell-adapted viruses (e.g., rotavirus [233]) are currently ongoing and demonstrating encouraging results [234]. Repurposing the virus vaccines for cancer immunotherapy is of particular interest since the pre-existing viral immunity may increase oncolytic effectiveness. Promising therapeutic outcomes in several clinical trials in various solid tumors inspire rapid advances of therapeutic approaches based on combining OVs with ICI and immunomodulatory molecules (as separate agents or OV payloads). We hope that the existing challenges of virotherapy will be resolved in the near future as the results of ongoing clinical trials appear.

## Figures and Tables

**Table 1:** Viral immunotherapy in clinical applications.

Combinatorial Agent	*n*	Function
Check-point inhibitors		
Pembrolizumab	14	Targets and blocks a PD-1 protein on the surface of T-cells. Blocking PD-1 triggers the T-cells activation towards finding and killing cancer cells. Pemrolizumab known under brand name Keytruda.
Nivolumab	8	An anti-PD-1 monoclonal antibody (brand name Opdivo).
Ipililumab	5	Humanized immune checkpoint inhibitor which blocks CTLA-4 receptor and upregulates cytotoxic T–lymphocytes (brand name Yervoy).
Avelumab	3	An anti-PD-1 monoclonal antibody (brand name Bavencio).
Durvalumab	3	An anti-PD-L1 specific human IgG1 kappa monoclonal antibody.
Cemiplimab	3	An anti-PD-1 monoclonal antibody (brand name Libtayo).
Atezolizumab	2	An anti-PDL-1 monoclonal antibody (brand name Tecentriq).
Socazolimab	1	Anti-PD-L1 monoclonal antibody (ZKAB001).
HX008	1	Anti-PD-L1 monoclonal antibody.
Vibostolimab	1	Vibostolimab is a monoclonal antibody against T-cell immunoreceptor with Ig and ITIM domains (TIGIT). Vibostolimab blocks the interaction between TIGIT and its ligands (CD112 and CD155) thereby activating T cells.
Bevacizumab	1	Bevacizumab (Avastin) targets cellular vascular endothelial growth factor (VEGF), a protein that is essential for blood vessel growth.
Trasuzumab	1	Trasuzumab (Herceptin) is an anti-HER2 monoclonal antibody targeting breast cancer and stomach cancer cells expressing HER2 receptors.
Tremelimumab	1	An anti- CTLA-4 monoclonal antibody.
Immunomodulatory factors		
Interleukine-2 (IL-2)	3	Stimulates cytotoxic T cells (CD8+) and NK cells, controls both the primary and secondary expansion of antigen-specific CD8+ T cell populations.
Interferon-α	3	Cytokine that activates immune cells (NK cells and T-cells) and suppresses tumor cell division by inhibiting protein and hormone synthesis. It also reduces angiogenesis through inhibition of angiogenic factors b-FGF and VEGF.
Granulocyte-macrophage colony-stimulating factor (GM-CSF)	3	GM-CSF enhances the number of circulating white blood cells and increases neutrophil and monocyte function. It also actively shapes the dendritic cell profile leading to enhanced anti-tumor effect.
Antigens		
Autologous tumor cells	14	Therapeutic agent produced from patient tumor cells. Processed and treated tumor cells are a great source of cancer antigens that, after administration, boost the immune system of the individual that they have been isolated.
Melanoma-associated antigen 3(MAGE-A3)	3	MAGE-A3 is a tumor-specific shared antigen often expressed in lung cancer and melanoma. Immunization with MAGE-A3 tends to stimulate the immune response to cancer, which has been traditionally considered as poorly immunogenic.
ag-E6E7	1	Human papillomavirus oncoproteins E6 and E7. Immunization with E6 and E7 antigens improves antitumor immunity against HPV-related tumors and enhances the immunogenicity of dendritic cells.
Bacillus Calmette-Guerin (BCG)	1	Nontumor antigen initially used as a tuberculosis vaccine. High immunogenic BCG mounts overall immune response that potentially decreases the reoccurrence of cancer.
Radiotherapy/Chemotherapy/Surgery	80	Various drugs, radiotherapy regimes accompanied by tumor resection (where possible) are in use in combination with virotherapy. The reader may find specific details in decent reviews and supplementary materials.
Single-agent virotherapy	88	Wild-type viruses attenuated or genetically engineered variants armored with immunomodulatory molecules are frequently used as a monotherapy. Variants of the used genetic modifications of oncolytics (mainly for stimulating the immune system) are shown in Table 2.

**Table 2:** Oncolytic viruses engineering and payloads in clinical practice.

Payload and Modifications	*n*	Function
GM-CSF (CSF2)	49	GM-CSF is a growth factor that stimulates differentiation, proliferation and migration of myeloid cells.
Thymidine kinase (TK)	24	HSV-1 TK is a virulence factor deletion of which attenuates virus, but not essential for virus replication. In addition, TK being used as a suicide gene to specifically target tumor cells.
Human sodium iodide symporter (hNIS)	14	NIS mediates a transport process of iodide uptake. Overexpression of NIS in cancer cells increases iodide concentration within the cells that benefit from radioiodine therapy.
p53 (TP53)	10	Tumor protein is a major tumor suppressor factor that acts through the regulation of the cell cycle. p53 is often malfunction in tumor cells.
Interferon β (IFN-beta)	8	IFN-beta is a cytokine, which has an antiviral and anti-proliferative effect. IFN-beta stimulates innate and adaptive immunity and has confirmed antitumor activity.
MAGE-A3	3	Tumor-specific antigen. MAGE-A3 immunization elicits antigen-specific immune response.
PSA-TRICOM (B7.1, ICAM-1, LFA-3)	2	Prostate-specific antigen (PSA). B7.1, ICAM-1, LFA-3 are T-cell costimulatory molecules.
Carcinoembryonic antigen (CEA)	2	CEA is a glycoprotein, which rarely found in the blood of adults. Expression of CEA serves as a marker for noninvasive monitoring of virus dissemination in vivo.
Interleukine-12 (IL-12)	1	IL-12 plays a central role in T-cell and natural killer cell responses, induces the production of interferon-γ (IFN-γ).
Fas-c and PPE-1 promoter	1	Chimeric death receptor Fas and TNF receptor 1 and modified endothelium-specific pre-proendothelin-1 (PPE-1) promoter delivered by virus vector may trigger apoptosis of endothelial cells and reduce tumor angiogenesis.
HPV E6/HPV E7	1	Human papillomavirus oncoproteins.
TERT promoter	1	Telomerase reverse transcriptase promoter (TERT) is used to attenuate virus replication.
Interferon-gamma (IFN-ɣ)	1	IFN-ɣ is a cytokine molecule with pronounced cytostatic, pro-apoptotic and immune-stimulating effects.
Tyrosinase-related protein (TYRP1)	1	TYRP1 is expressed in melanomas and on the surface of melanocytes and is an immunoreactive protein.
Anti-CTLA4	1	blocks CTLA-4 receptor and upregulate cytotoxic T –lymphocytes
None	109	Many wild-type viruses have an oncolytic potential and are frequently used without payload. Attenuated or evolutionary selected viruses also demonstrate strong antitumor effect.

## Supplementary Materials

The following supplementary material (Table S1) is available online at https://www.mdpi.com/article/10.3390/v13071271/s1.
