# Supplementary material for: Combinatorial Approaches for Cancer Treatment Using Oncolytic Viruses: Projecting the Perspectives through Clinical Trials Outcomes
Source: Viruses. 2021 Jun 29;13(7):1271. doi: 10.3390/v13071271 (PMC8309967; doi:10.3390/v13071271)
Supplement: Supplementary file 1 [file viruses-13-01271-s001.zip › viruses-1260010-supplementary.pdf]

Table S1.

Virus	Oncolytic virus variant	Payload	Monotherapy / Combination	Combo agent 1	Combo agent 2	Combo agent 3	Type of Cancer	Patients (n)	Phase (1, 1/2, 2, 3)	Route	Clinical trial (ID)	Reference
Adenovirus	TG1042 (Ad-IFN-gamma)	IFN-gamma	Mono	NO	NO	NO	Cutaneous T cell lymphoma	13	2	IT	NCT00394693	doi.org/10.1371/journal.pone.0083670
Adenovirus	Ad5/3-D24-GMCSF (CGTG-102)	GM-CSF	Mono	NO	NO	NO	Soft-tissue sarcoma (STS) or primary bone sarcoma	15	1/2	IT	N/A	doi.org/10.1002/ijc.28696
Adenovirus	rAd-p53 (Gendicine)	p53	Combo	Radiotherapy	NO	NO	Hepatocellular carcinoma	40	N/A	IT	N/A	doi.org/10.1007/s00432-009-0701-6
Adenovirus	rAd-p53 (Gendicine)	p53	Combo	Chemotherapy	NO	NO	Stage III or IV oral carcinoma who had refused or were ineligible	99	3	Intra-arterial	ChiCTR-TRC-09000392	doi.org/10.1186/1741-7015-12-16 ChiCTR-TRC-09000392
Adenovirus	rAd-p53 (Gendicine)	p53	Combo	Chemotherapy	NO	NO	Locally advanced cervical cancer (LACC)	40	N/A	IT	N/A	doi.org/10.3892/ol.2017.5901
Adenovirus	rAd-p53 (Gendicine)	p53	Combo	Chemotherapy	NO	NO	Malignant pleural effusion induced by lung cancer	35	N/A	IT	N/A	doi.org/10.5732/cjc.009.10149
Adenovirus	rAd-p53 (Gendicine)	p53	Combo	Surgery	NO	NO	Laryngeal cancer	12	1	IT	N/A	PMID: 14703409
Adenovirus	rAd-p53 (Gendicine)	p53	Combo	Radiotherapy	NO	NO	Head and neck squamous cell carcinoma	36	N/A	IT	N/A	PMID: 16188130
Adenovirus	rAd-p53 (Gendicine)	p53	Combo	Radiotherapy	NO	NO	Nasopharyngeal carcinoma	29	2	IT	N/A	PMID: 14703410
Adenovirus	rAd-p53 (Gendicine)	p53	Combo	Radiotherapy	NO	NO	Head and neck squamous cell carcinoma	42	2	IT	N/A	PMID: 14703408
Adenovirus	rAd-p53 (Gendicine)	p53	Combo	Surgery	NO	NO	Recurrent malignant gliomas	38	N/A	IT	N/A	PMID: 21122390
Adenovirus	rAd-p53 (Gendicine)	p53	Combo	Chemotherapy	NO	NO	Recurrent nasopharyngeal carcinoma	162	1	IT	N/A	doi.org/10.1097/CAD.0000000000000448
Adenovirus	Human adenovirus type 5	NO	Combo	Chemotherapy	NO	NO	Unresectable hepatocellular carcinoma	266	3	Intra-arterial	NCT01869088	doi.org/10.1007/s12032-014-0095-4
Adenovirus	OBP-401	TERT promoter + EIA and EIB	Mono	NO	NO	NO	Newly diagnosed esophageal, stomach, colon, liver, pancreatic, prostate, endometrial or cervical cancer without preoperative chemotherapy or radiotherapy	86	Observational	NA	NCT04064515	doi.org/10.3892/or.2014.3436
Adenovirus	Human adenovirus type 5 Sitimagene ceradenovec	CD/HSV-1 TK	Combo	Chemotherapy	Ganciclovir	NO	Operable high-grade primary or recurrent glioma	250	3	IT	EudraCT Number 2004-000464-28	doi.org/10.1016/S1470-2045(13)70274-2
Adenovirus	VB-111	Fas-c + PPE-1 promoter	Combo	Chemotherapy	NO	NO	Platinum-resistant ovarian cancer	21	1/2	IV	NCT01711970	doi.org/10.1177/1947601912437933 doi.org/10.1016/j.ygyno.2020.02.034
Adenovirus	Human adenovirus type 5 (Telomelysin)	hTERT	Mono	NO	NO	NO	Advanced Solid Tumors	16	1	IT	N/A	doi.org/10.1038/mt.2009.262
Adenovirus	ICOVIR-7	NO	Mono	NO	NO	NO	Advanced and refractory solid tumors	21	N/A	NA	N/A	doi.org/10.1158/1078-0432.CCR-09-3167
Adenovirus	Ad5/3-D24-GMCSF (CGTG-102)	GM-CSF	Mono	NO	NO	NO	Advanced cancer	60	N/A	NA	N/A	doi.org/10.1158/1078-0432.CCR-12-2546
Adenovirus	Ad5/ 3-E2F-Δ24-GMCSF (CGTG-602)	GM-CSF + E2F1	Combo	Chemotherapy	NO	NO	Advanced metastatic tumors - Ovarian Rectum Colon Pancreatic Melanoma Breast Sarcoma Fibrosarcoma cancer	13	1	IT	N/A	doi.org/10.18632/oncotarget.2901
Adenovirus	Enadenotucirev (ColoAd1) Ad11p/Ad3 chimera	NO	Mono	NO	NO	NO	Colorectal cancer , NSCLC, urothelial cell cancer, renal cell cancer	17	1	IT	NCT02053220	doi.org/10.1186/s40425-017-0277-7
Adenovirus	Enadenotucirev (ColoAd1) Ad11p/Ad3 chimera	NO	Mono	NO	NO	NO	Solid tumors of Epithelial Origin, Metatstatic colorectal cancer	61	1/2	IV	NCT02028442	doi.org/10.1186/s40425-019-0510-7
Adenovirus	Enadenotucirev (ColoAd1) Ad11p/Ad3 chimera	NO	Combo	Chemoradiotherapy	NO	NO	Locally advanced rectal cancer	30	1	IV	NCT03916510	doi.org/10.1186/s13014-020-01593-5
Adenovirus	DNX-2401 (Delta-24-RGD) RGD motif + E1A 24bp deletion	NO	Mono	NO	NO	NO	Diffuse intrinsic pontine glioma	1	1	IT	EudraCT: 2016-001577-33	doi.org/10.3389/fonc.2018.00061 doi.org/10.1093/neuros/nyx507
Adenovirus	DNX-2401 (Delta-24-RGD) RGD motif + E1A 24bp deletion	NO	Combo	Chemotherapy	NO	NO	Brain cancer	37	1	IT	NCT00805376	doi.org/10.1200/JCO.2017.75.8219
Adenovirus	Autologous MSCs Carrying the Oncolytic Virus Icovir-5 (Celyvir) d-DM-E2F-K-Delta24RGD	NO	Combo	Autologous MSC	NO	NO	Solid tumors	20	1	IV	NCT01844661	doi.org/10.1016/j.ymthe.2020.01.019
Adenovirus	Ad5-CD/TKrep	CD/HSV-1 TK	Combo	Chemotherapy	Radiotherapy	Valganciclovir	Prostate cancer	15	1	IT	N/A	PMID: 14612551
Adenovirus	Ad5-CD/TKrep	CD/HSV-1 TK	Mono	NO	NO	NO	Prostate cancer	16	1	IT	N/A	PMID: 12208748
Adenovirus	Ad5-CD/TKrep	CD/HSV-1 TK	Mono	NO	NO	NO	Ovarian cancer	38	1	Intrapleural	NCT02028117	N/A
Adenovirus	Ad5-CD/TKrep	CD/HSV-1 TK	Combo	Nivolumab	NO	NO	Colorectal cancer	135	1	NA	NCT02636036	N/A
Poxvirus	Pexa-Vec (JX-594)	GM-CSF + TK-del + lac-Z gene under p7.5 promoter	Mono	NO	NO	NO	Neuroblastoma, rhabdomyosarcoma, lymphoma	6	1	IT	NCT01169584	doi.org/10.1038/mt.2014.243
Poxvirus	Pexa-Vec (JX-594)	GM-CSF + TK-del + lac-Z gene under p7.5 promoter	Mono	NO	NO	NO	HCC	16	2	IV	NCT01636284	N/A
Poxvirus	Pexa-Vec (JX-594)	GM-CSF + TK-del + lac-Z gene under p7.5 promoter	Mono	NO	NO	NO	Unresectable malignant melanoma	10	1/2	IT	NCT00429312	N/A
Poxvirus	Pexa-Vec (JX-594)	GM-CSF + TK-del + lac-Z gene under p7.5 promoter	Combo	Chemotherapy	NO	NO	HCC	25	2	IV+IT	NCT01171651	N/A
Poxvirus	Pexa-Vec (JX-594)	GM-CSF + TK-del + lac-Z gene under p7.5 promoter	Mono	NO	NO	NO	HCC	129	2	NA	NCT01387555	N/A

Poxvirus	Pexa-Vec (JX-594)	GM-CSF + TK-del + lac-Z gene under p7.5 promoter	Combo	Chemotherapy	NO	NO	Metastatic, refractory colorectal carcinoma	52	1/2	IV	NCT01394939	N/A
Poxvirus	Pexa-Vec (JX-594)	GM-CSF + TK-del + lac-Z gene under p7.5 promoter	Mono	NO	NO	NO	Colorectal Cancer	15	1	IV	NCT01380600	doi.org/10.1038/mt.2015.109
Poxvirus	Pexa-Vec (JX-594)	GM-CSF + TK-del + lac-Z gene under p7.5 promoter	Mono	NO	NO	NO	Refractory primary or metastatic liver cancer - hepatocellular, colorectal, melanoma, and lung cancer	14	1	TD	NCT00629759	doi.org/10.1016/S14702045(08)70107-4
Poxvirus	Pexa-Vec (JX-594)	GM-CSF + TK-del + lac-Z gene under p7.5 promoter	Mono	NO	NO	NO	Refractory Solid Tumors	23	1	IV	NCT00625456	N/A
Poxvirus	Pexa-Vec (JX-594)	GM-CSF + TK-del + lac-Z gene under p7.5 promoter	Combo	Chemotherapy	Avelumab	NO	Solid Tumors	197	1/2	IV	NCT02630368	N/A
Poxvirus	Pexa-Vec (JX-594)	GM-CSF + TK-del + lac-Z gene under p7.5 promoter	Mono	NO	NO	NO	Colorectal carcinoma	2	2	IV	NCT01329809	N/A
Poxvirus	Pexa-Vec (JX-594)	GM-CSF + TK-del + lac-Z gene under p7.5 promoter	Mono	NO	NO	NO	Unresectable primary HCC	30	2	IT	NCT00554372	doi.org/10.1007/978-1-4939-2727-2_19, 10.1038/nm.3089
Poxvirus	Pexa-Vec (JX-594)	GM-CSF + TK-del + lac-Z gene under p7.5 promoter	Mono	NO	NO	NO	Refractory renal cell carcinoma	17	2	IV	N/A	doi.org/10.1200/JCO.2018.36.6_suppl.671
Poxvirus	Pexa-Vec (JX-594)	GM-CSF + TK-del + lac-Z gene under p7.5 promoter	Combo	Nivolumab	NO	NO	HCC	30	1/2	IT	NCT03071094	N/A
Poxvirus	Pexa-Vec (JX-594)	GM-CSF + TK-del + lac-Z gene under p7.5 promoter	Combo	ZKAB001 (anti-PD-L1 monoclonal)	NO	NO	Local progression or metastatic melanoma with failed first-line treatment	40	1/2	IT	NCT04849260	N/A
Poxvirus	Pexa-Vec (JX-594)	GM-CSF + TK-del + lac-Z gene under p7.5 promoter	Combo	Durvalumab	Tremelimumab	NO	Colorectal Cancer	34	1/2	IV	NCT03206073	N/A
Poxvirus	Pexa-Vec (JX-594)	GM-CSF + TK-del + lac-Z gene under p7.5 promoter	Combo	Cemiplimab	NO	NO	Renal cell carcinoma	117	1/2	IV/IT	NCT03294083	N/A
Poxvirus	Pexa-Vec (JX-594)	GM-CSF + TK-del + lac-Z gene under p7.5 promoter	Combo	Ipilimumab	NO	NO	Advanced solid tumors	66	1/2	IT	NCT02977156	N/A
Poxvirus	Pexa-Vec (JX-594)	GM-CSF + TK-del + lac-Z gene under p7.5 promoter	Combo	Chemotherapy	NO	NO	HCC	459	3	IT	NCT02562755	doi.org/10.1200/JCO.2016.34.15_suppl.TPS4146
Poxvirus	JX-963	GM-CSF	Mono	NO	NO	NO	Prostate cancer	83	1	IV	N/A	doi.org/10.1172/JCI32727
Poxvirus	GL-ONC1 F14.5L, J2R (encoding thymidine kinase) + A56R (encoding hemagglutinin) loc	NO	Combo	Chemotherapy	Bevacizumab	NO	Ovarian cancer, peritoneal carcinomatosis, fallopian tube cancer	64	1/2	IP	NCT02759588	N/A
Poxvirus	GL-ONC1 F14.5L, J2R (encoding thymidine kinase) + A56R (encoding hemagglutinin) loc	NO	Mono	NO	NO	NO	Solid organ cancers	5	1	IV	NCT02714374	N/A
Poxvirus	GL-ONC1 F14.5L, J2R (encoding thymidine kinase) + A56R (encoding hemagglutinin) loc	NO	Mono	NO	NO	NO	Advanced Stage Cancer (Solid tumor disease), AML	10	N/A	NA	NCT03420430	N/A
Poxvirus	GL-ONC1 F14.5L, J2R (encoding thymidine kinase) + A56R (encoding hemagglutinin) loc	NO	Mono	NO	NO	NO	Advanced solid tumors	43	1	IV	NCT00794131	N/A
Poxvirus	GL-ONC1 F14.5L, J2R (encoding thymidine kinase) + A56R (encoding hemagglutinin) loc	NO	Mono	NO	NO	NO	Peritoneal carcinomatosis	9	1	IP	NCT01443260	doi.org/10.1158/1078-0432.CCR-18-0244
Poxvirus	GL-ONC1 F14.5L, J2R (encoding thymidine kinase) + A56R (encoding hemagglutinin) loc	NO	Mono	NO	NO	NO	Lung Cancer	18	1	IT	NCT01766739	N/A
Poxvirus	GL-ONC1 F14.5L, J2R (encoding thymidine kinase) + A56R (encoding hemagglutinin) loc	NO	Combo	Chemotherapy	Radiotherapy	NO	HNC, non-metastatic	19	1	IV	NCT01584284	doi.org/10.1158/1078-0432.CCR-16-3232
Poxvirus	vvDD (delVGF + TK mutation)	NO	Mono	NO	NO	NO	Advanced solid tumors	26	1	IV/IT	NCT00574977	doi.org/10.1038/mt.2014.194, 10.1038/mt.2016.101
Poxvirus	vvDD (delVGF + TK mutation)	NO	Combo	autologous cytokine induced killer cells.	NO	NO	Advanced solid tumors	24	1	NA	NCT04282044	N/A
Poxvirus	DryVax	NO	Mono	NO	NO	NO	Bladder cancer, carcinoma	4	1	Intravesical	N/A	PMID: 11547060 . J Urol. 2001; 166(4): 1291–1295
Poxvirus	ACAM2000	NO	Mono	NO	NO	NO	Advanced solid tumors, AML	26	1	IV/IT	N/A	doi.org/10.1200/JCO.2019.37.8_suppl.39
Poxvirus	PROSTVAC-V/F TRICOM	PSA-TRICOM	Combo	GM-CSF	Flutamide	NO	Prostate cancer	64	2	SC	NCT00450463	N/A
Poxvirus	PROSTVAC-V/F TRICOM	PSA-TRICOM	Combo	GM-CSF	NO	NO	Prostate cancer	1297	3	SC	NCT01322490	doi.org/10.1200/JCO.18.02031
Herpes Simplex Virus	T-VEC γ34.5 and α47 deletions	GM-CSF	Mono	NO	NO	NO	Melanoma	31	3	IT	NCT01368276	N/A
Herpes Simplex Virus	T-VEC γ34.5 and α47 deletions	GM-CSF	Mono	NO	NO	NO	Melanoma	50	2	IT	NCT00289016	N/A

Herpes Simplex Virus	T-VEC<br>γ34.5 and α47 deletions	GM-CSF	Mono	NO	NO	NO	Melanoma	26	1	IT	N/A	doi.org/10.1158/1078-0432.CCR-06-0759
Herpes Simplex Virus	T-VEC<br>γ34.5 and α47 deletions	GM-CSF	Mono	NO	NO	NO	Melanoma	437	3	IT	NCT00769704	doi.org/10.1200/JCO.2014.58.3377<br>doi.org/10.1186/s40425-019-0623-z<br>doi.org/10.1186/s40425-017-0276-8
Herpes Simplex Virus	T-VEC<br>γ34.5 and α47 deletions	GM-CSF	Combo	Ipilimumab	NO	NO	Melanoma	217	1/2	IT	NCT01740297	doi.org/ 10.1200/JCO.2017.73.7379<br>doi.org/ 10.1007/s00262-017-1967-1<br>doi.org/ 10.1200/JCO.2016.67.1529
Herpes Simplex Virus	T-VEC<br>γ34.5 and α47 deletions	GM-CSF	Combo	Pembrolizumab	NO	NO	Melanoma	713	3	IT	NCT02263508	doi.org/ 10.1038/s41416-019-0530-6<br>doi.org/ 10.1007/s00262-017-1967-1<br>doi.org/ 10.1016/j.cell.2017.08.027
Herpes Simplex Virus	T-VEC<br>γ34.5 and α47 deletions	GM-CSF	Combo	BRAF and MEK inhibitors	NO	NO	Melanoma	4	1	IT	NCT03088176	N/A
Herpes Simplex Virus	T-VEC<br>γ34.5 and α47 deletions	GM-CSF	Combo	Chemotherapy	NO	NO	Melanoma	32	3	IT	NCT02288897	N/A
Herpes Simplex Virus	T-VEC<br>γ34.5 and α47 deletions	GM-CSF	Combo	Radiotherapy	NO	NO	Melanoma	19	2	IT	NCT02819843	N/A
Herpes Simplex Virus	T-VEC<br>γ34.5 and α47 deletions	GM-CSF	Combo	Chemotherapy	Radiotherapy	NO	Squamous Cell Carcinoma, HNC	5	3	IT	NCT01161498	N/A
Herpes Simplex Virus	T-VEC<br>γ34.5 and α47 deletions	GM-CSF	Mono	NO	NO	NO	Melanoma	3	2	IT	NCT02574260	N/A
Herpes Simplex Virus	T-VEC γ34.5 and α47 deletions	GM-CSF	Mono	NO	NO	NO	Pancreatic cancer	17	1	IT	NCT00402025	N/A
Herpes Simplex Virus	T-VEC<br>γ34.5 and α47 deletions	GM-CSF	Combo	Nivolumab	NO	NO	Stage IV metastatic cancer, lung cancer	1	1/2	IT	NCT03597009	N/A
Herpes Simplex Virus	T-VEC<br>γ34.5 and α47 deletions	GM-CSF	Combo	Chemotherapy	NO	NO	Tripple Negative breast cancer	50	1/2	IT	NCT02779855	N/A
Herpes Simplex Virus	T-VEC<br>γ34.5 and α47 deletions	GM-CSF	Mono	NO	NO	NO	Non-melanoma Skin Cancer, Basal cell Carcinoma,<br>Cutaneous Lymphoma	20	1	IT	NCT03458117	N/A
Herpes Simplex Virus	T-VEC<br>γ34.5 and α47 deletions	GM-CSF	Mono	NO	NO	NO	Melanoma	61	2	IT	NCT02014441	N/A
Herpes Simplex Virus	T-VEC<br>γ34.5 and α47 deletions	GM-CSF	Mono	NO	NO	NO	Malignant Chest Wall Neoplasm,<br>Recurrent Breast Carcinoma	11	2	IT	NCT02658812	N/A
Herpes Simplex Virus	T-VEC<br>γ34.5 and α47 deletions	GM-CSF	Mono	Nivolumab	NO	NO	Refractory lymphomas or Advanced<br>or refractory Non-melanoma skin cancer	68	2	IT	NCT02978625	N/A
Herpes Simplex Virus	Oncolytic Type 2 Herpes Simplex Virus	GM-CSF	Combo	Chemotherapy	HX008	NO	Solid tumors	300	1/2	IT	NCT03866525	doi.org/10.1136/jitc-2020-002224
Herpes Simplex Virus	Oncolytic Type 2 Herpes Simplex Virus	GM-CSF	Combo	Pembrolizumab	NO	NO	Solid tumors	30	1/2	IT	NCT04386967	N/A
Herpes Simplex Virus	OrienX010	GM-CSF	Mono	NO	NO	NO	Melanoma	30	1	IT	NCT03048253	doi.org/10.1093/annonc/mdx667.001
Herpes Simplex Virus	OrienX010	GM-CSF	Mono	NO	NO	NO	Melanoma, Liver Cancer, Pancreatic cancer, Lung Cancer	18	1	IT	NCT01935453	N/A
Herpes Simplex Virus	OrienX010	GM-CSF	Mono	NO	NO	NO	Melanoma	30	1	IV	NCT04206358	N/A
Herpes Simplex Virus	NV1020, γ-34.5, ICP0, ICP4, and LAT genes have been deleted	NO	Mono	NO	NO	NO	Colorectal cancer, metastatic cancer	13	1/2	Intra-arterial	NCT00012155	doi.org/10.1016/S1525-0016(16)39150-X<br>doi.org/10.1089/hum.2010.020
Herpes Simplex Virus	ONCR-177	tissue-specific miRNA, UL37 mutation + IL12, FLT3LG, CCL4, PD-1 and CTLA-4.	Combo	Pembrolizumab	NO	NO	Melanoma, Solid Tumors,<br>head and neck squmous cell carcinoma,<br>Breast cancer	132	1	IT	NCT04348916	N/A
Herpes Simplex Virus	HF-10 (canerpaturev) attenuated HSV-1	NO	Mono	NO	NO	NO	Solid tumors	6	1	IT	NCT02428036	N/A
Herpes Simplex Virus	HF-10 (canerpaturev) attenuated HSV-1	NO	Mono	NO	NO	NO	Solid tumors	28	1	IT	NCT01017185	N/A
Herpes Simplex Virus	HSV1716, ICP34.5 deletion	NO	Mono	NO	NO	NO	Rhabdomyosarcoma, Osteosarcoma,<br>Ewing Sarcoma, Neuroblastoma	18	1	IV/IT	NCT00931931	doi.org/10.1158/1078-0432.CCR-16-2900
Herpes Simplex Virus	HSV1716, ICP34.5 deletion	NO	Mono	NO	NO	NO	Colorectal cancer, Liver Neoplasma	32	1/2	Intra-arterial	NCT00149396	N/A
Herpes Simplex Virus	HSV1716, ICP34.5 deletion	NO	Mono	NO	NO	NO	Malignant Pleural Mesothelioma	12	1/2	Intrapleural	NCT01721018	N/A
Herpes Simplex Virus	G207, HSV-1 mutant R3616 and γ34.5 deletion	NO	Combo	Radiotherapy	NO	NO	Glioma, Astrocytoma, Glioblastoma	65	1/2	IT	NCT00028158	N/A
Herpes Simplex Virus	G207, HSV-1 mutant R3616 and γ34.5 deletion	NO	Mono	NO	NO	NO	Malignant glioma	9	1	IT	NCT00157703	doi.org/10.1038/mt.2014.22
Herpes Simplex Virus	G207, HSV-1 mutant R3616 and γ34.5 deletion	NO	Combo	Radiotherapy	NO	NO	Supratentorial Neoplasms, Glioma ,<br>Glioblastoma, Astrocytoma	12	1	IT	NCT02457845	doi.org/10.1056/NEJMoa2024947
Herpes Simplex Virus	T-VEC<br>γ34.5 and α47 deletions	GM-CSF	Combo	Ipilimumab	Nivolumab	NO	Breast Cancer	6	1	IT	NCT04185311	N/A
Herpes Simplex Virus	Oncolytic Type 2 Herpes Simplex Virus	GM-CSF	Mono	NO	NO	NO	Pancreatic cancer	25	1/2	IT	NCT04637698	N/A
Herpes Simplex Virus	HSV-1, RP-2-001-18	anti-CTLA-4	Combo	Nivolumab	NO	NO	Solid Tumors	36	1	IT	NCT04336241	N/A
Herpes Simplex Virus	HSV-1, RP-1	GALV-GP R- protein and GM-CSF.	Combo	Cemiplimab	NO	NO	Squamous Cell cancer	240	2	IT	NCT04050436	N/A
Herpes Simplex Virus	HSV-1, RP-2-001-18	GALV-GP R- protein and GM-CSF.	Combo	Nivolumab	NO	NO	Melanoma, NSCLC, Non-melanoma Skin Cancer	300	2	IT	NCT03767348	doi.org/10.1200/JCO.2020.38.15_suppl.e22050
Herpes Simplex Virus	G47D<br>deletion of the a47 gene, γ34.5 gene	NO	Mono	NO	NO	NO	Prostate cancer	9	1	NA	UMIN000010463	N/A

Vesicular Stomatitis virus	VSV-IFNB -NIS	IFN-beta + hNIS	Combo	Avelumab	NO	NO	Refractory solid tumours, metastatic colorectal cancer, pheochromocytoma, neuroendocrine tumors	114	1	IV/IT	NCT02923466	doi.org/10.1200/JCO.2020.38.15_suppl.3090
Vesicular Stomatitis virus	VSV-rIFNB	IFN-beta + hNIS	Mono	NO	NO	NO	Hepatocellular cancer and tumours with metastatic lesions in the liver	17	1	IT	NCT01628640	N/A
Vesicular Stomatitis virus	VSV-IFNB -NIS	IFN-beta + hNIS	Combo	Pembrolizumab	NO	NO	Solid Tumors, HNSCC, NSCLC	142	1/2	IV	NCT03647163	N/A
Vesicular Stomatitis virus	VSV-IFNbetaTYRP1	IFN-beta + TYRP1	Mono	NO	NO	NO	Melanoma	72	1	IV/IT	NCT03865212	N/A
Vesicular Stomatitis virus	VSV-IFNB -NIS	IFN-beta + hNIS	Combo	Chemotherapy	NO	NO	Recurrent Endometrial Cancer	77	1	IV	NCT03120624	doi.org/10.1158/1538-7445.AM2018-CT072
Vesicular Stomatitis virus	VSV-IFNB -NIS	IFN-beta + hNIS	Combo	Chemotherapy	NO	NO	Relapsed or refractory MM, AML, T-cell Lymphoma	65	1	IV	NCT03017820	https://ash.confex.com/ash/2020/webprogram/Paper140853.html
Vesicular Stomatitis virus	VSV-IFNB -NIS	IFN-beta + hNIS	Combo	Cemiplimab	NO	NO	Melanoma, HCC, NSCLC, endometrial cancer	152	2	IV/IT	NCT04291105	N/A
Vesicular Stomatitis virus	VSV-IFNB -NIS	IFN-beta + hNIS	Combo	18F-TFB-PET	NO	NO	Myeloma Before MV-NIS Treatment, Endometrial Cancer Before VSV-hINF-NIS Treatment	2	1	IV	NCT03456908	N/A
Maraba virus (Vesiculovirus)	MG1 Maraba/MAGE-A3	MAGE-A3	Combo	MAGE-A3 (AdMA3)	NO	NO	Incurable MAGE-A3-expressing solid tumours	56	1	IV	NCT02285816	N/A
Maraba virus (Vesiculovirus)	MG1 Maraba/MAGE-A3	MAGE-A3	Combo	MAGE-A3 (AdMA3)	Pembrolizumab	NO	NSCLC	16	1	IV	NCT02879760	N/A
Maraba virus (Vesiculovirus)	MG1-E6E7	HPV E6 + HPV E7	Combo	Ad-E6E7	Atezolizumab	NO	HPV Associated Cancers	75	1	IV	NCT03618953	N/A
Maraba virus (Vesiculovirus)	MG1 Maraba/MAGE-A3	MAGE-A3	Combo	MAGE-A3 (AdMA3)	Pembrolizumab	Cyclophosphamide	Previously treated metastatic melanoma or cutaneous squamous cell carcinoma	0	1	IV/IT	NCT03773744	N/A
Reovirus	Reolysin® (pelareorep, Reovirus Serotype-3-dearing Strain)	NO	Mono	NO	NO	NO	Sarcoma	53	2	IV	NCT00503295	N/A
Reovirus	Reolysin® (pelareorep, Reovirus Serotype-3-dearing Strain)	NO	Combo	Chemotherapy	NO	NO	Recurrent malignant glioma	18	1	IT	NCT00528684	doi.org/10.1038/mt.2014.21
Reovirus	Reolysin® (pelareorep, Reovirus Serotype-3-dearing Strain)	NO	Mono	NO	NO	NO	Fallopian tube carcinoma ovarian carcinoma, primary peritoneal carcinoma	70	1	IV	NCT00602277	N/A
Reovirus	Reolysin® (pelareorep, Reovirus Serotype-3-dearing Strain)	NO	Mono	NO	NO	NO	Metastatic melanoma	23	2	IV	NCT00651157	doi.org/ 10.1038/mt.2012.146
Reovirus	Reolysin® (pelareorep, Reovirus Serotype-3-dearing Strain)	NO	Combo	Carboplatin	Paclitaxel	NO	Head and neck cancer	14	2	IV	NCT00753038	N/A
Reovirus	Reolysin® (pelareorep, Reovirus Serotype-3-dearing Strain)	NO	Combo	Carboplatin	Paclitaxel	NO	Carcinoma, NSCLC	37	2	IV	NCT00861627	N/A
Reovirus	Reolysin® (pelareorep, Reovirus Serotype-3-dearing Strain)	NO	Combo	Carboplatin	Paclitaxel	NO	Metastatic melanoma	14	2	IV	NCT00984464 REO 020	10.1038/mt.2012.146 28289863 10.1007/s00280-017-3260-6
Reovirus	Reolysin® (pelareorep, Reovirus Serotype-3-dearing Strain)	NO	Combo	Carboplatin	Paclitaxel	NO	Squamous cell carcinoma of the lung	32	2	IV	NCT00998192	N/A
Reovirus	Reolysin® (pelareorep, Reovirus Serotype-3-dearing Strain)	NO	Combo	Gemcitabine	NO	NO	Metastatic pancreatic adenocarcinoma	34	2	IV	NCT00998322	N/A
Reovirus	Reolysin® (pelareorep, Reovirus Serotype-3-dearing Strain)	NO	Combo	Chemotherapy	NO	NO	Carcinoma, squamous cell of the Head and Neck	167	3	IV	NCT01166542	10.1586/ecp.12.53
Reovirus	Reolysin® (pelareorep, Reovirus Serotype-3-dearing Strain)	NO	Combo	Chemotherapy	NO	NO	Recurrent ovarian, tubal or peritoneal cancer	108	2	IV	NCT01199263	doi.org/10.1016/j.ygyno.2017.07.135
Reovirus	Reolysin® (pelareorep, Reovirus Serotype-3-dearing Strain)	NO	Combo	Chemotherapy	NO	NO	Childhood Solid tumor	26	1	IV	NCT01240538	N/A
Reovirus	Reolysin® (pelareorep, Reovirus Serotype-3-dearing Strain)	NO	Combo	Chemotherapy	Bevacizumab	Leucovorin	KRAS mutant metastatic colorectal cancer	36	1	IV	NCT01274624	N/A
Reovirus	Reolysin® (pelareorep, Reovirus Serotype-3-dearing Strain)	NO	Combo	Chemotherapy	NO	NO	Metastatic pancreatic adenocarcinoma	73	2	IV	NCT01280058	doi.org/10.1038/mt.2016.66
Reovirus	Reolysin® (pelareorep, Reovirus Serotype-3-dearing Strain)	NO	Mono	NO	NO	NO	Relapsed MM	12	1	IV	NCT01533194	doi.org/10.1158/1078-0432.CCR-14-1404
Reovirus	Reolysin® (pelareorep, Reovirus Serotype-3-dearing Strain)	NO	Combo	Docetaxel	Prednisone	NO	Prostate cancer	85	2	IV	NCT01619813	N/A
Reovirus	Reolysin® (pelareorep, Reovirus Serotype-3-dearing Strain)	NO	Combo	Bevacizumab	FOLFOX6	NO	Metastatic colorectal cancer	109	2	IV	NCT01622543	doi.org/10.1016/j.clcc.2018.03.001
Reovirus	Reolysin® (pelareorep, Reovirus Serotype-3-dearing Strain)	NO	Combo	Chemotherapy	NO	NO	Metastatic breast cancer	81	2	IV	NCT01656538 CCTG IND213	doi.org/ 10.1007/s10549-017-4538-4
Reovirus	Reolysin® (pelareorep, Reovirus Serotype-3-dearing Strain)	NO	Combo	Chemotherapy	NO	NO	Advanced or metastatic NSCLC	166	2	IV	NCT01708993	doi.org/10.1016/j.lungcan.2018.03.005
Reovirus	Reolysin® (pelareorep, Reovirus Serotype-3-dearing Strain)	NO	Combo	Carfilzomib|	Dexamethasone	NO	Refractory MM	28	1	IV	NCT02101944	N/A
Reovirus	Reolysin® (pelareorep, Reovirus Serotype-3-dearing Strain)		Combo	Bortezomib	Dexamethasone	NO	Plasma cell myeloma	14	1	IV	NCT02514382	N/A
Reovirus	Reolysin® (pelareorep, Reovirus Serotype-3-dearing Strain)	NO	Combo	Chemotherapy	Pembrolizumab	NO	Pancreatic ductal adenocarcinoma	11	1	IV	NCT02620423	doi.org/10.1158/1078-0432.CCR-19-2078
Reovirus	Reolysin® (pelareorep, Reovirus Serotype-3-dearing Strain)	NO	Combo	Chemotherapy	NO	NO	Refractory MM	4	1	IV	NCT03015922	N/A
Reovirus	Reolysin® (pelareorep, Reovirus Serotype-3-dearing Strain)	NO	Combo	Nivolumab	Carfilzomib	Dexamethasone	Recurrent plasma cell myeloma	62	1	IV	NCT03605719	N/A
Reovirus	Reolysin® (pelareorep, Reovirus Serotype-3-dearing Strain)	NO	Combo	Pembrolizumab	NO	NO	Pancreatic adenocrcinoma	17	2	IV	NCT03723915	N/A
Reovirus	Reolysin® (pelareorep, Reovirus Serotype-3-dearing Strain)	NO	Combo	Trastuzumab	Atezolizumab	Letrozole	Breast cancer	38	1	IV	NCT04102618	N/A

Reovirus	Reolysin® (pelareorep, Reovirus Serotype-3-dearing Strain)	NO	Combo	Avelumab	Paclitaxel	NO	Breast cancer	48	2	IV	NCT04215146	N/A
Reovirus	Reolysin® (pelareorep, Reovirus Serotype-3-dearing Strain)	NO	Mono	NO	NO	NO	Breast cancer	25	2	IV	NCT04445844	N/A
Reovirus	Reolysin® (pelareorep, Reovirus Serotype-3-dearing Strain)	NO	Combo	GM-CSF	NO	NO	Brain tumors (glioma)	6	1	IV	NCT02444546	N/A
Reovirus	Reolysin® (pelareorep, Reovirus Serotype-3-dearing Strain)	NO	Mono	NO	NO	NO	Brain tumors (malignant gliomas)	12	1/2	IT	N/A	doi.org/10.1038/sj.mt.6300403
Reovirus	Reolysin® (pelareorep, Reovirus Serotype-3-dearing Strain)	NO	Mono	NO	NO	NO	Solid tumors	19	1	IT	REO-001	doi.org/10.1007/s10637-012-9865-z
Reovirus	Reolysin® (pelareorep, Reovirus Serotype-3-dearing Strain)	NO	Combo	Chemotherapy	NO	NO	Advanced or metastatic solid tumors refractory to standard of care treatment	25	1	IV	REO-010	doi.org/10.1158/1078-0432.CCR-10-1233
Reovirus	Reolysin® (pelareorep, Reovirus Serotype-3-dearing Strain)	NO	Combo	Chemotherapy	NO	NO	NSCLC, Colorectal, Breast, Cervical, Squamous cell carcinoma, Undifferentiated carcinoma, Poorly differentiated carcinoma, Cholangiocarcinoma, Oesophageal adenocarcinoma, Fibrosarcoma	16	1	IV	N/A	doi.org/10.1158/1078-0432.CCR-10-2159
Reovirus	Reolysin® (pelareorep, Reovirus Serotype-3-dearing Strain)	NO	Mono	NO	NO	NO	Advanced solid tumors	18	1	IV	N/A	doi.org/10.1007/s10637-009-9279-8
Reovirus	Reolysin® (pelareorep, Reovirus Serotype-3-dearing Strain)	NO	Combo	Radiotherapy	NO	NO	Solid tumors	23	1	IT	N/A	doi.org/10.1158/1078-0432.CCR-10-0054 doi.org/10.1016/j.cytogfr.2010.02.006
Reovirus	Reolysin® (pelareorep, Reovirus Serotype-3-dearing Strain)	NO	Combo	Chemotherapy	NO	NO	Extra-cranial solid tumors, refractory solid tumors	29	1	IV	N/A	doi.org/10.1002/pbc.25464
Reovirus	Reolysin® (pelareorep, Reovirus Serotype-3-dearing Strain)	NO	Combo	Chemotherapy	NO	NO	SCCHN, HNC, melanoma, gynecologic cancer	31	1/2	IV	N/A	doi.org/10.1158/1078-0432.CCR-11-2181
Reovirus	Reolysin® (pelareorep, Reovirus Serotype-3-dearing Strain)	NO	Combo	Chemotherapy	Radiotherapy	NO	Head and neck, prostate, colorectal, pancreas, melanoma, soft-tissue sarcoma, bladder cancer, NSCLC, renal cancer, endometrial cancer	33	1	IV	N/A	doi.org/10.1158/1078-0432.CCR-08-0524
Reovirus	Reolysin® (pelareorep, Reovirus Serotype-3-dearing Strain)	NO	Combo	Chemotherapy	NO	NO	Solid tumors	36	1	IV	N/A	doi.org/10.1158/1078-0432.CCR-14-1770
Enterovirus	Coxsackie Virus A21, CAVATAK, CAV21, V937	NO	Combo	Pembrolizumab	NO	NO	Melanoma	36	1	IT	NCT02565992	N/A
Enterovirus	Coxsackie Virus A21, CAVATAK, CAV21, V937	NO	Combo	Mitomycin C	NO	NO	Non-muscle invasive bladder cancer	16	1	Intravesical	NCT02316171	PMID: 31273010 doi.org/10.1158/1078-0432.CCR-18-4022
Enterovirus	Coxsackie Virus A21, CAVATAK, CAV21, V937	NO	Combo	Ipilimumab	NO	NO	Melanoma	50	1	IT	NCT02307149	https://www.medpagetoday.com/reading-room/asco/melanoma/66052
Enterovirus	Coxsackie Virus A21, CAVATAK, CAV21, V937	NO	Mono	NO	NO	NO	Melanoma	16	2	IT	NCT01636882	N/A
Enterovirus	Coxsackie Virus A21, CAVATAK, CAV21, V937	NO	Combo	Ipilimumab	NO	NO	Uveal melanoma, liver metastases	11	1	IV	NCT03408587	N/A
Enterovirus	Coxsackie Virus A21, CAVATAK, CAV21, V937	NO	Mono	NO	NO	NO	Stage IV Melanoma	9	1	IT	NCT00438009	N/A
Enterovirus	Coxsackie Virus A21, CAVATAK, CAV21, V937	NO	Mono	NO	NO	NO	Melanoma, breast cancer, prostate cancer	8	1	IV	NCT00636558	N/A
Enterovirus	Coxsackie Virus A21, CAVATAK, CAV21, V937	NO	Mono	NO	NO	NO	Malignant melanoma	57	2	IT	NCT01227551	N/A
Enterovirus	Coxsackie Virus A21, CAVATAK, CAV21, V937	NO	Combo	Pembrolizumab	NO	NO	NSCLC, bladder cancer	85	1	IV	NCT02043665	https://cancerres.aacrjournals.org/content/77/13_Supplement/CT115
Enterovirus	Coxsackie Virus A21, CAVATAK, CAV21, V937	NO	Combo	Pembrolizumab	NO	NO	Neoplasm metastasis	185	1/2	IT	NCT04521621	N/A
Enterovirus	Coxsackie Virus A21, CAVATAK, CAV21, V937	NO	Combo	Pembrolizumab	NO	NO	Advanced, metastatic melanoma	135	2	IV/IT	NCT04152863	N/A
Enterovirus	Coxsackie Virus A21, CAVATAK, CAV21, V937	NO	Combo	Pembrolizumab	Vibostolimab	NO	Melanoma	65	1/2	IT	NCT04303169	N/A
Enterovirus	Polyovirus (chimeric polio-rhino viruses - PVSRIPO)	NO	Mono	NO	NO	NO	Invasive breast cancer	6	1	IT	NCT03564782	N/A
Enterovirus	Polyovirus (chimeric polio-rhino viruses - PVSRIPO)	NO	Mono	NO	NO	NO	Malignant glioma	122	2	IT	NCT02986178	N/A
Enterovirus	Polyovirus (chimeric polio-rhino viruses - PVSRIPO)	NO	Mono	NO	NO	NO	Unresectable melanoma	18	1	IT	NCT03712358	doi.org/10.1136/jitc-2020-002203
Enterovirus	Polyovirus (chimeric polio-rhino viruses - PVSRIPO)	NO	Mono	NO	NO	NO	Melanoma	56	2	IT	NCT04577807	N/A
Enterovirus	Polyovirus (chimeric polio-rhino viruses - PVSRIPO)	NO	Combo	Pembrolizumab	NO	NO	Glioblastoma, recurrent glioblastoma, supratentorial glioblastoma, brain tumors	30	2	IT	NCT04479241	N/A

Enterovirus	Polyovirus (chimeric polio-rhino viruses - PVSRIPO)	NO	Mono	NO	NO	NO	Malignant glioma, anaplastic sstrocytoma, anaplastic oligoastrocytoma, anaplastic oligodendroglioma, glioblastoma, gliosarcoma, atypical reratoid/rhabdoid tumor of brain, medulloblastoma, ependymoma, pleomorphic xanthoastrocytoma of brain, embryonal tumor of brain	12	1	IT	NCT03043391	N/A
Enterovirus	Polyovirus (chimeric polio-rhino viruses - PVSRIPO)	NO	Mono	NO	NO	NO	Glioma, glioblastoma, malignant glioma, GBB	61	1	IT	NCT01491893	doi.org/10.1158/2159-8290.CD-NB2018-098
Newcastle disease virus	NDV (73T strain)	NO	Combo	Autologous tumor cell lysate	NO	NO	Stage II metastatic melanoma (AJCC stage III)	83	2	NA	N/A	doi.org/10.1007/BF02987752; PMID: 9990864
Newcastle disease virus	NDV (73T strain)	NO	Combo	Autologous tumor cell lysate	IL-2	IFN-alpha	Locally advanced renal cell carcinoma	208	2	NA	N/A	doi.org/10.1007/BF00184874
Newcastle disease virus	An attenuated oncolytic veterinary vaccine strain (MTH-68/H)	NO	Mono	NO	NO	NO	Various advanced chemorefractory cancers, lung metastases	59	2	Inhalation	NCT00348842	PMID: 8275514
Newcastle disease virus	Oncolytic veterinary vaccine strain (MTH-68/H)	NO	Mono	NO	NO	NO	GBM, high-grade glioma	4	N/A	NA	N/A	doi.org/10.1023/b:neon.0000021735.85511.05
Newcastle disease virus	NDV (HUJ strain)	NO	Mono	NO	NO	NO	GBM	14	1/2	IV	NCT01174537	doi.org/ 10.1016/j.ymthe.2005.08.016
Newcastle disease virus	NDV-modified autologous melanoma cell lysate	NO	Combo	Autologous tumor cell lysate	IL-2	NO	Melanoma	29	1	Intradermal	N/A	doi.org/10.1046/j.1610-0387.2003.02014.x
Newcastle disease virus	NDV (PV701 strain)	NO	Mono	NO	NO	NO	Advanced solid tumors	79	1	IV	N/A	doi.org/ 10.1200/JCO.2002.08.042
Newcastle disease virus	NDV (PV701 strain)	NO	Mono	NO	NO	NO	Advanced solid tumors	18	1	IV	N/A	doi.org/ 10.1158/1078-0432.CCR-06-1817
Newcastle disease virus	NDV (PV701 strain)	NO	Mono	NO	NO	NO	Incurable solid tumors	16	1	IV	N/A	doi.org/10.1158/1078-0432.CCR-05-2038
Newcastle disease virus	Autologous tumor cell vaccine ATV-NDV	NO	Combo	Autologous tumor cells	NO	NO	Colorectal carcinoma Dukes'	16	1	Intradermal	N/A	doi.org/10.1002/1097-0142(19901001)66:7<1517::aid-cncr2820660714>3.0.co;2-i
Newcastle disease virus	Autologous tumor cell vaccine ATV-NDV	NO	Combo	Autologous tumor cells	NO	NO	Early breast cancer, metastatic breast cancer, metastatic ovarian cancer	121	1	Intradermal	N/A	doi.org/10.1200/JCO.1997.15.4.1354
Newcastle disease virus	Autologous tumor cell vaccine ATV-NDV	NO	Combo	Autologous tumor cells	IL-2	NO	HNSCC	20	1	Intradermal	N/A	doi.org/10.1158/0008-5472.CAN-04-1545
Newcastle disease virus	Autologous tumor cell vaccine ATV-NDV (avirulent strain Ulster of NDV)	NO	Combo	Autologous tumor cells	Bacillus Calmette Gudrin (BCG)	NO	Colorectal resected carcinoma	57	2	Intradermal	N/A	PMID: 9816085
Newcastle disease virus	Autologous tumor cell vaccine ATV-NDV	NO	Combo	Autologous tumor cells	IFN-alpha	NO	Renal cell cancer patients	40	2	Intradermal	N/A	doi.org/10.3892/ijo.6.5.947
Newcastle disease virus	Autologous tumor cell vaccine ATV-NDV	NO	Combo	Autologous tumor cells	NO	NO	GBM	111	2	Intradermal	N/A	doi.org/10.1200/JCO.2004.09.038
Newcastle disease virus	Autologous tumor cell vaccine ATV-NDV	NO	Combo	Autologous tumor cells	NO	NO	Colorectal cancer	50	3	Intradermal	N/A	doi.org/10.1007/s00262-008-0526-1 doi.org/10.1586/14760584.2014.854169
Newcastle disease virus	Autologous tumor cell vaccine ATV-NDV	NO	Combo	Autologous tumor cells	NDV-specific biAbs	NO	Colorectal resected carcinoma	51	1	Intradermal	N/A	doi.org/10.1007/s00262-008-0526-1
Newcastle disease virus	MEDI5395	GM-CSF	Combo	Durvalumab	NO	NO	Advanced solid tumors	188	1	NA	NCT03889275	doi.org/10.1158/1538-7445.AM2020-CT244
Newcastle disease virus	MEDI9253	IL-12	Combo	Durvalumab	NO	NO	Solid tumors	86	1	NA	NCT04613492	N/A
Measles virus	The unmodified commercially available MV-Edm-Zagreb vaccine strain (MV-EZ)	NO	Combo	IFN-alpha	NO	NO	Cutaneous T-cell lymphoma IIb or higher, resistant to or relapsing after conventional therapies	5	1	IT	N/A	doi.org/10.1182/blood-2004-11-4558
Measles virus	MV-CEA virus (oncolytic MV-Edm derivatives genetically engineered to expressed the human carcinoembryonic antigen)	CEA	Mono	NO	NO	NO	Taxol- and platinum-resistant ovarian cancer	21	1	IP	N/A	doi.org/10.1158/0008-5472.CAN-09-2762
Measles virus	MV-NIS virus (oncolytic MV-Edm derivatives genetically engineered to expressed the human sodium iodide symporter)	hNIS	Combo	Chemotherapy	NO	NO	Relapsed and refractory MM	32	1	IV	N/A	doi.org/10.1038/leu.2017.120
Measles virus	MV-NIS virus (oncolytic MV-Edm derivatives genetically engineered to expressed the human sodium iodide symporter)	hNIS	Mono	NO	NO	NO	Taxol- and platinum-resistant ovarian cancer	16	1	IP	N/A	doi.org/10.1158/0008-5472.CAN-14-2533
Measles virus	MV-NIS virus (oncolytic MV-Edm derivatives genetically engineered to expressed the human sodium iodide symporter)	hNIS	Mono	NO	NO	NO	Ovarian, fallopian or peritoneal Cancer	66	2	IP	NCT02364713	doi.org/10.2174/1568009617666170222125035
Measles virus	MV-NIS virus (oncolytic MV-Edm derivatives genetically engineered to expressed the human sodium iodide symporter)	hNIS	Combo	Autologous MSC	NO	NO	Recurrent ovarian, primary peritoneal or fallopian tube cancer	57	1/2	IP	NCT02068794	N/A
Measles virus	MV-NIS virus (oncolytic MV-Edm derivatives genetically engineered to expressed the human sodium iodide symporter)	hNIS	Mono	NO	NO	NO	Malignant peripheral nerve sheath tumor	30	1	IT	NCT02700230	N/A
Measles virus	MV-NIS virus (oncolytic MV-Edm derivatives genetically engineered to expressed the human sodium iodide symporter)	hNIS	Mono	NO	NO	NO	Malignant pleural mesothelioma	15	1	Intrapleural	NCT01503177	N/A

Measles virus	MV-NIS virus (oncolytic MV-Edm derivatives genetically engineered to expressed the human sodium iodide symporter)	hNIS	Mono	NO	NO	NO	Recurrent/Metastatic SCC of the head and neck, metastatic breast cancer	12	1	IT	NCT01846091	N/A
Measles virus	MV-CEA virus (oncolytic MV-Edm derivatives genetically engineered to expressed the human carcinoembryonic antigen)	CEA	Mono	NO	NO	NO	GBM	23	1	IT	NCT00390299	N/A
Others (Parvovirus , SVV)	Seneca Valley Virus (SVV-001, NTX-010)	NO	Mono	NO	NO	NO	Advanced solid tumors with neuroendocrine features, SCLC	30	1	IV	NCT00314925	doi.org/10.2147/OV.S96915
Others (Parvovirus , Seneca Valley virus)	Seneca Valley Virus (SVV-001, NTX-010)	NO	Combo	Chemotherapy	NO	NO	Pediatric patients with neuroblastoma, rhabdomyosarcoma, rare tumors with NET feature	22	1	IV	NCT01048892	doi.org/10.2147/OV.S96915
Others (Parvovirus , SVV)	Seneca Valley Virus (SVV-001, NTX-010)	NO	Mono	NO	NO	NO	Patients with SCLC who did not progress after platinum-based chemotherapy	26	2	IV	NCT01017601	doi.org/10.1016/j.jtho.2019.09.083
Others (Parvovirus , SVV)	H-1PV (ParvOryx®)	NO	Mono	NO	NO	NO	Glioblastoma	18	1/2	IT	NCT01301430	doi.org/10.1016/j.ymthe.2017.08.016
Others (Parvovirus , SVV)	H-1PV (ParvOryx®)	NO	Mono	NO	NO	NO	Pancreatic carcinoma	7	1/2	IV/IT	NCT02653313	N/A
